# Multi-omics analyses unveil dual genetic loci governing four distinct watermelon flesh color phenotypes

**DOI:** 10.1186/s43897-025-00166-y

**Published:** 2025-05-14

**Authors:** Na Li, Shilai Xing, Gaofei Sun, Jianli Shang, Jia-Long Yao, Nannan Li, Dan Zhou, Yu Wang, Yuan Lu, Jinpeng Bi, Jiming Wang, Hongfeng Lu, Shuangwu Ma

**Affiliations:** 1 National Key Laboratory for Germplasm Innovation & Utilization of Horticultural Crops, Zhengzhou Fruit Research Institute, Chinese Academy of Agricultural Sciences, Zhengzhou, 450009 China; 2 Zhongyuan Research Center, Chinese Academy of Agricultural Sciences, Xinxiang, 453500 China; 3 Berry Genomics Corporation, Beijing, 100015 China; 4 School of Computer Science and Information Engineering, Anyang Institute of Technology, Anyang, 455000 China; 5 The New Zealand Institute for Plant and Food Research Limited, Auckland, 1025 New Zealand

**Keywords:** Fruit flesh color, Carotenoid biosynthesis, Gene structural variation, Chromoplast development, Marker-assisted breeding, *Citrullus lanatus* (watermelon)

## Abstract

Watermelon fruit flesh displays various colors. Although genetic loci underlying these variations are identified, the molecular mechanism remains elusive. Here, we assembled a chromosome-scale reference genome of an elite watermelon and developed integrated genetic maps using single nucleotide polymorphism (SNP) and structural variation markers. Several key genetic varients for fruit shape and flesh color were identified. Two variants associated with flesh color were further studied, including one copy number variant (CNV, a triplicate of 1.2 kb DNA) in the promoter region of *REDUCED CHLOROPLAST COVERAGE 2* (*ClREC2*) and one SNP in *Lycopene β-Cyclase* (*ClLCYB*) coding region*.* These two variants together explained 99.7% of the flesh color variations in 314 watermelon accessions. The SNP in *ClLCYB* was the same as previously reported, disrupting ClLCYB function. The CNV could strongly enhance *ClREC2* expression, consequently increasing the expression of carotenoid biosynthesis genes, the number of plastoglobules within chromoplasts, and carotenoid level in mature fruit flesh. Finally, we proposed a “two-switch” genetic model by integrating two major causative loci, which can explain the formation of the four main flesh colors in different watermelon accessions. These results provide new insights into the regulation of carotenoid biosynthesis and color formation in plants.

**Supplementary Information:**

The online version contains supplementary material available at 10.1186/s43897-025-00166-y.

## Core 

Through systematic integration of dual genetic loci, we confirmed the molecular mechanism underlying watermelon flesh color differentiation. Four phenotypically distinct flesh colors in various watermelon accessions arise through coordinated action of two regulators: *Lycopene β-Cyclase* (*ClLCYB*) determines which type of carotenoid (yellow vs. red), while *REDUCED CHLOROPLAST COVERAGE 2* (*ClREC2*) modulates the level of red carotenoid (coral red vs. scarlet red). The new insights offer valuable guidance and advance our understanding of carotenoid biosynthesis and accumulation in plants.

## Gene & accession numbers

All sequence data and genome files generated for this study were deposited in the NCBI Sequence Read Archive (SRA) under BioProject PRJNA982968, PRJNA980840, PRJNA983768 and PRJNA983085.

## Introduction

Watermelon is a major horticultural crop grown throughout temperate regions of the world. It exhibits wide diversity in fruit traits. The favourable fruit traits to watermelon growers and sellers are include good rind hardness, which strongly impacts transportability, storability and shelf life (Liao et al. 2020). Flesh color, fruit shape, rind color and rind stripe pattern of watermelon are also important fruit quality traits to filfull consumers’ acceptance (Guo et al. 2019). However, breeding for stacking multiple desirable traits is challenging because molecular mechanisms underlying many traits are unclear.

Previous studies on watermelon fruit traits were focused on sugar content (Ren et al. 2018; Ren et al. 2020; Ren et al. 2021), fruit size (Ren et al. 2014; Sandlin et al. 2012; Li et al. 2023), fruit shape (Li et al. 2021; Dou et al. 2018; Legendre et al. 2020) and flesh color (Guo et al. 2019; Zhang et al. 2020; Liu et al. 2022; Li et al. 2020; Bang et al. 2007; Branham et al. 2017). However, the genetic and molecular bases of most traits are still elusive. Taking fruit flesh color as an example, watermelon accessions exhibit extreme diversity in this trait, including white, pale-yellow, canary-yellow, salmon-yellow, orange and red (coral red and scarlet red) colors. Although several genetic loci, including *B*, *C*, *i-C*, *Wf*, *y*, *y*^*O*^, *Y*^*Scr*^ and *Y*^*crl*^ (Wehner 2012), have been reported to control flesh color, only one gene, the lycopene β-cyclase *ClLCYB* gene on chromosome 4 has mapped to flesh color locus using accessions with different flesh colors, such as white versus red (Zhang et al. 2020) and pale or canary yellow versus red (Bang et al. 2007). The flesh color is determined by composition and concentration of different carotenoids accumulated in the flesh. Lycopene is red while xanthophylls is yellow. A single nucleotide polymorphism (SNP) mutation in *ClLCYB* reduces ClLCYB protein abundance, block the convertion from lycopene to β-carotene, increases lycopene level, and thus confers a red flesh phenotype (Zhang et al. 2020). Furthermore, a quantitative trait locus (QTL) on chromosome 6, *Y*^*sc*r^, was shown to confer a deep red color, scarlet red, fruit flesh (Li et al. 2020) and another locus at the same genomic region on chromosome 6 controls white flesh (Yi et al. 2023). It is difficult to accurately phenotype scarlet red vs. coral red in segregating populations because the colors are similar. This difficulty presents challanges to the identification of the gene underpining the scarlet red phenotype. Without the information on the gene and casuasive variants for each locus, it is impossible to fully understand the relationships among these loci in controlling different flesh colors.

Gene structural variations (SVs), including copy number variants (CNVs), insertions/delections (InDels), duplications and inversions, are widely present in genomes (Alkan et al. 2011). SVs explain phenotypic variation as much as or more than SNPs do (Chaisson et al. 2019). In plants, SVs regulate important traits such as male sterility in rice (Xu et al. 2022), plant architecture in cotton (Ji et al. 2021), fruit shape in tomato (Xiao et al. 2008), and sex determination in melon (Martin et al. 2009). However, comprehensive SV catalogs are rare and are beginning to appear in rice (Fuentes et al. 2019) and maize (Sun et al. 2018). Genome-wide identification of SVs has generally lagged behind that of SNPs because the lack of high-quality reference genomes and high-depth whole-genome resequencing of many samples (Wang et al. 2018). Although gene presence/absence variations were mentioned based on a *Citrullus* genus super-pangenome (Wu et al. 2023), genome-wide identification of SVs for watermelon has not been reported till now.

To fill a major gap in precisely identifying the key genetic variants underlying fruit traits in watermelon, we de novo assembled a high quality reference genome of an elite watermelon cultivar with coral red flesh, and then constructed integrated genetic maps using SNP and SV markers generated from deep resequencing of core watermelon collections. These newly generated resources were used to identify a SV underlying the difference (between scarlet red and coral red flesh) in watermelon. The resources and knowledge generated in this study would be invaluable for many further studies.

## Results

### De novo genome assembly and gene annotation

A de novo assembly of the DR117 genome with coral red flesh was achieved by using PacBio long sequence reads and a Bionano optical genetic map based on direct label and stain technology. The initial assembly using PacBio long reads and Illumina short reads (Table S1) generated 104 contigs with an N50 length of 28.28 Mb. These contigs were assembled into 84 scaffolds using Bionano optical mapping data (Table S2) and subsequently corrected with Illumina sequences. The scaffolds showed an N50 size of 35.2 Mb and a cumulative length of 371.8 Mb (Table S3) and included 11 superscaffolds corresponding to the 11 chromosomes, each with a length greater than 25 Mb, accounting for 98.88% of the assembled genome (Figure S1) (Guo et al. 2019; Wu et al. 2019). The estimated genome size was 418 Mb according to 19-mer depth distribution analysis (Figure S2), which was lower than that reported in earlier analyses (Wu et al. 2019; Guo et al. 2013; Arumuganathan and Earle 1991). The contiguity of the DR117 assembly was similar to that of G42 (Deng et al. 2022), but much greater than that of 97103v2 (Guo et al. 2019) and Charleston Gray (Wu et al. 2019) (Table S3). BUSCO analysis (Simao et al. 2015) revealed that 92.3% of the core eukaryotic genes were present in the watermelon genome. A total of 24,839 protein-coding genes were predicted (Table S4), which was similar as those in G42 (24,205), but was substantially greater than other previous assemblies (22,596 for 97103v2 and 22,549 for WCG) (Guo et al. 2019; Wu et al. 2019; Deng et al. 2022).

### Flesh color and carotenoid biosynthesis genes are related to the geographic differentiation

To identify genetic variants underpinning fruit fresh color evolution, the genome of 196 *Citrullus* accessions was sequenced. These accessions represented a *Citrullus* core collection, which included one *C. naudinianus*, one *C. rehmii*, one *C. ecirrhosus*, nine *C. colocynthis*, 34 *C. amarus*, 21 *C. mucosospermus*, and 129 *C. lanatus* accessions (Table S5), capturing 97.5% of the allele diversity in the collection of 1,022 watermelon accessions (Figure S3, Table S6). A total of 2.47 Tb of sequence data were generated using 150 bp paired-end sequence, with an average depth of 30.1X (Table S5). In total, 24,497,491 SNPs were identified, with an average of 66.6 SNPs per kilobase, of which 1,050,977 SNPs were in coding regions, causing 557,073 missense variations in 23,303 genes. Based on these SNPs, a phylogenetic tree was constructed (Figure S4), showing a tree structure largely consistent with that reported in previous studies (Guo et al. 2019; Renner et al. 2017). One accession (PI 490375) of the wild species *C. mucosospermus*, collected from Mali (East Africa), was most closely related to the cultivated species *C. lanatus*, and grouped into the same clade with two *C. lanatus* accessions PI 226506 and Koresta (Figure S4). Notably, *C. lanatus* accessions were clearly separated into four groups, each group showed closely related geographical distribution.

**Fig. 1:**
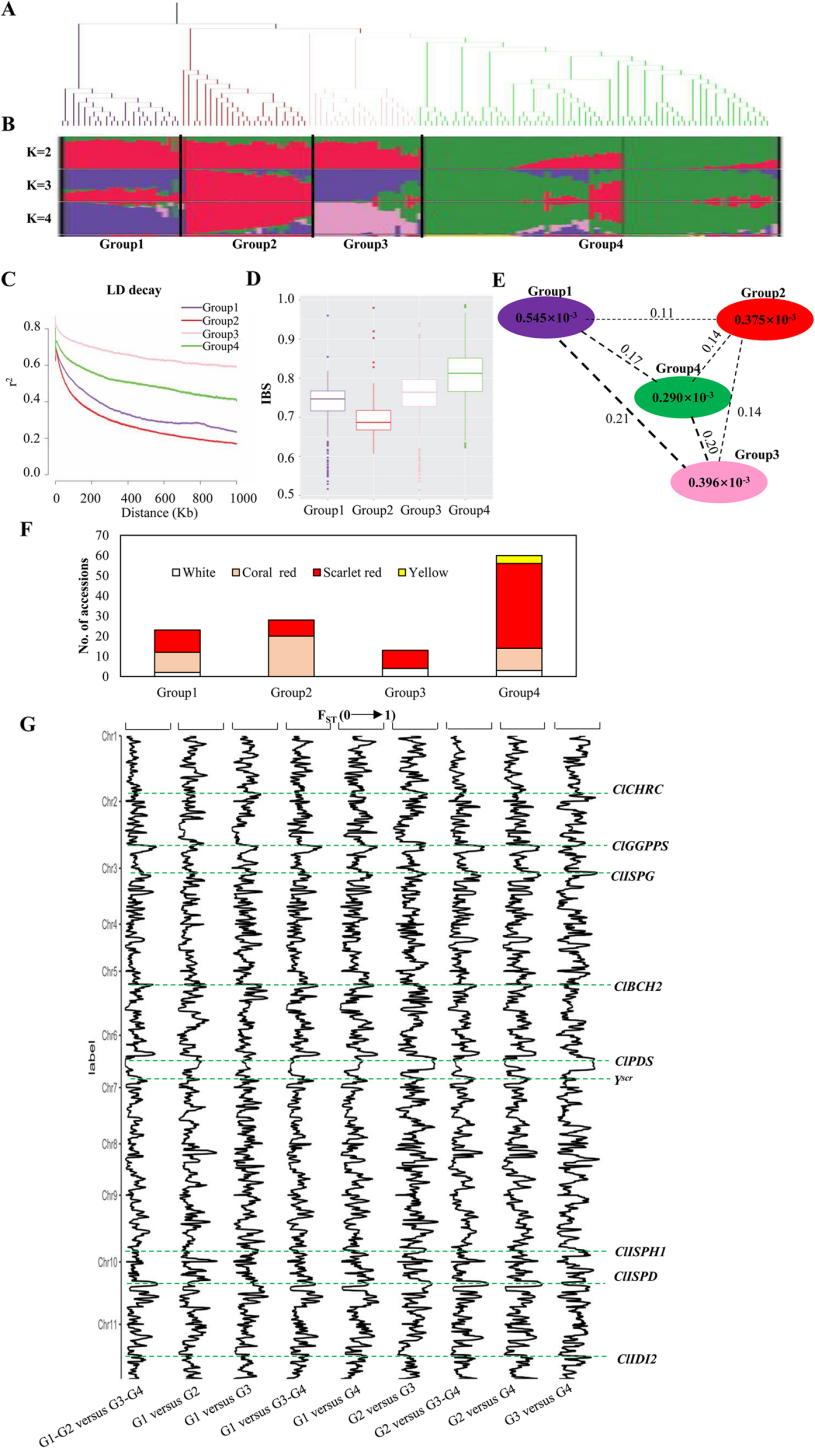
Population diversity and selective sweep analysis. **A** Phylogenetic tree was constructed based on genome-wide SNPs. The four cultivated watermelon accessions were classified into four subgroups, Group 1, Group 2, Group 3 and Group 4. **B** Population structure of 129 accessions with K = 2, 3, and 4. **C** Decay of LD in each group. **D** IBS for each group. **E** θπ and Fst among the four groups. The value in each circle indicates the level of θπ for each group, and the value on each line represents the Fst between the two groups. **F** Distribution of different flesh colors in the different groups. **G** Fst values across the entire genome for pairwise group comparisons. The green dashed line indicates the locations of genes

To better understand the genetics of watermelon improvement resulted from modern breeding, we performed phylogenetic relationship and population structure analyses using the 129 accessions of *C. lanatus* (Fig. 1). The inference drawn from phylogenetic analysis (Fig. 1A) and population structure (Fig. 1B) supported the classification of *C. lanatus* accessions into four groups. Group 1 comprised landraces and cultivars mainly from west-south China and Turkey; Group 2 comprised landraces and cultivars mainly from America; Group 3 comprised landraces and cultivars mainly from northern China; and Group 4 was biased toward cultivars from southern China or Japan. When K = 2, the main ancestor component of Group 4 was split, indicating that Group 4 had the highest level of selection. When K = 3, the main ancestor component of Group 2 was split. When K = 4, the main ancestor components of Group 1 and Group 3 were split (Fig. 1B). These results indicated that geographical distribution of these accessions was closely related to the group classification. Varieties with similar domestication levels tended to have more similar genetic backgrounds. The nucleotide diversities (θπ) (0.545 × 10^–3^) was the highest for Group 1, followed by Group 3 (0.396 × 10^–3^), Group 2 (0.375 × 10^–3^), and Group 4 (0.290 × 10^–3^). This pattern was opposite to that of linkage disequilibrium (LD) decay and identical-by-state (IBS) values (Fig. 1C, D). The lowest Fst value (0.11) was observed for Group 1 versus Group 2 (Fig. 1E), which is consistent with the similar climatic conditions for west-south China and America. Group 4 consisted of cultivars derived from southern China and Japan, which had the highest level of selection and gene exchange.

In addition to SNP analysis, genome-wide SV analysis was performed by leveraging the high-depth genome sequence data. A total of 11,620 SVs, ranging from 27 bp to 6 Mb, were identified, including 6,023 deletions, 795 duplications, 65 inversions, and 4,737 translocations (Table S7). Most Group 2 accessions had coral red fleshed fruits, while most Group 3 and Group 4 had scarlet red fleshed fruits (Fig. 1F). Pairwise F_ST_ analysis between different groups of *C. lanatus* revealed divergent peaks co-located with the genes encoding key metabolic enzymes of the methylerythritol 4-phosphate (MEP) and the carotenoid biosynthesis (CB) pathway (Fig. 1G, Table S8). Four MEP genes, 2-C-methyl-D-erythritol 4-phosphate cytidylyltransferase (*ClISPD*), 4-hydroxy-3-methylbut-2-en-1-yl diphosphate synthase (*ClISPG*), 4-hydroxy-3-methylbut-2-enyl diphosphate reductase (*ClISPH1*) and isopentenyl-diphosphate delta-isomerase I (*ClIDI2*) were selected in Group 3 and Group 4. Three CB genes, geranylgeranyl diphosphate synthase (*ClGGPPS*), which overlaps with the epistatic *Wf* gene for flesh color (Jie et al. 2014), phytoene desaturase (*ClPDS*) and β-carotene hydroxyl-lase (*ClBCH2*) was selected in Group 3 and Group 4. A plastoglobulin carotenoid-associated protein (*ClCHRC*), which regulates carotenoid sequestration and storage and enhances the accumulation of carotenoids (Kilambi et al. 2013), was selected in Group 3. Notably, the flesh color locus *Y*^*sc*r^ (Li et al. 2020), was selected in Group3 and Group 4. In addition, the selective sweeps for flesh color overlapped with the corresponding genome-wide association study (GWAS) signals (Table S9).

### GWAS identified genetic variants underpinning fruit shape and flesh color variation

SNP-GWAS (Figure S5A, Table S9) and SV-GWAS (Figure S5B, Table S9) showed that a SNP with a C/T polymorphism (S3_29499779) and an InDel with a length of 159 bp (InDel3_29499687) were strongly associated with fruit shape. Both variants were in the third exon of the *ClSUN* gene of different accessions (Figure S5C). It is known that SUN controls fruit shape in cucurbits and tomato (Xiao et al. 2008; Pan et al. 2020). These variants were the same as previously reported by QTL analysis of different segregating populations (Li et al. 2021; Dou et al. 2018; Legendre et al. 2020). Compared to the DR117 reference genome (with spheroidal fruit), the sequences of the lines with elongated fruits had a 159 bp deletion in exon 3 from 663 to 882 bp or a C to T mutation in exon 3 at 727 bp (Figure S5C). The point mutation was a nonsynonymous resulting in an amino acid change from aspartic acid to asparagine. To confirm the association between these variants and fruit shape, sequencing data of 370 accessions, included the 196 core accessions in present study and 174 accessions in previous study (Guo et al. 2019), was analyzed. Accessions being homozygous for the T SNP or for the 159 bp deletion showed elongated fruit, except for 20 accessions (Table S10). These two variants explained 94.59% of the phenotypic variation. This result was confirmed by analyzing of 193 accessions using a CAPS marker previously developed (Li et al. 2021), which could detect both variants simultaneously (Table S10).

To analysis the evolution history of fruit shape, these two variants were analyzed in 196 core accessions representing different watermelon species. The result summarized in Table S11 showed that 33 accessions of five wild species (1 *C. naudinianus*, 1 *C. rehmii*, 1 *C. ecirrhosus,* 9 *C. colocynthis* and 21 *C. mucosospermus*) had the spheroidal fruit shape and wild-type alleles of both variants S3_29499779 (C/C) and InDel3_29499687 (without the 159 deletions). This result indicated that the mutation might not happen in these wild species. From the wild species *C. amaus,* 34 accessions were analyzed, none had mutation at InDel3_29499687, but 16 accessions had mutation at S3_29499779. However, only 8 of them showed elongated fruit, indicating other unknow factors might prevent elongate fruit development. From the cultivated species *C. lanatus,* 129 accessions were analyzed, 103 had no mutation at either variation site, 10 had mutation at S3_29499779, another 16 had mutation at InDel3_29499687. One accession had mutation at InDel3_29499687, but had no elongated fruit, whereas two accession had no mutation at either site, but had elongated fruit. Once again, this result suggests other unknow factors affecting fruit shape of watermelon.

**Fig. 2:**
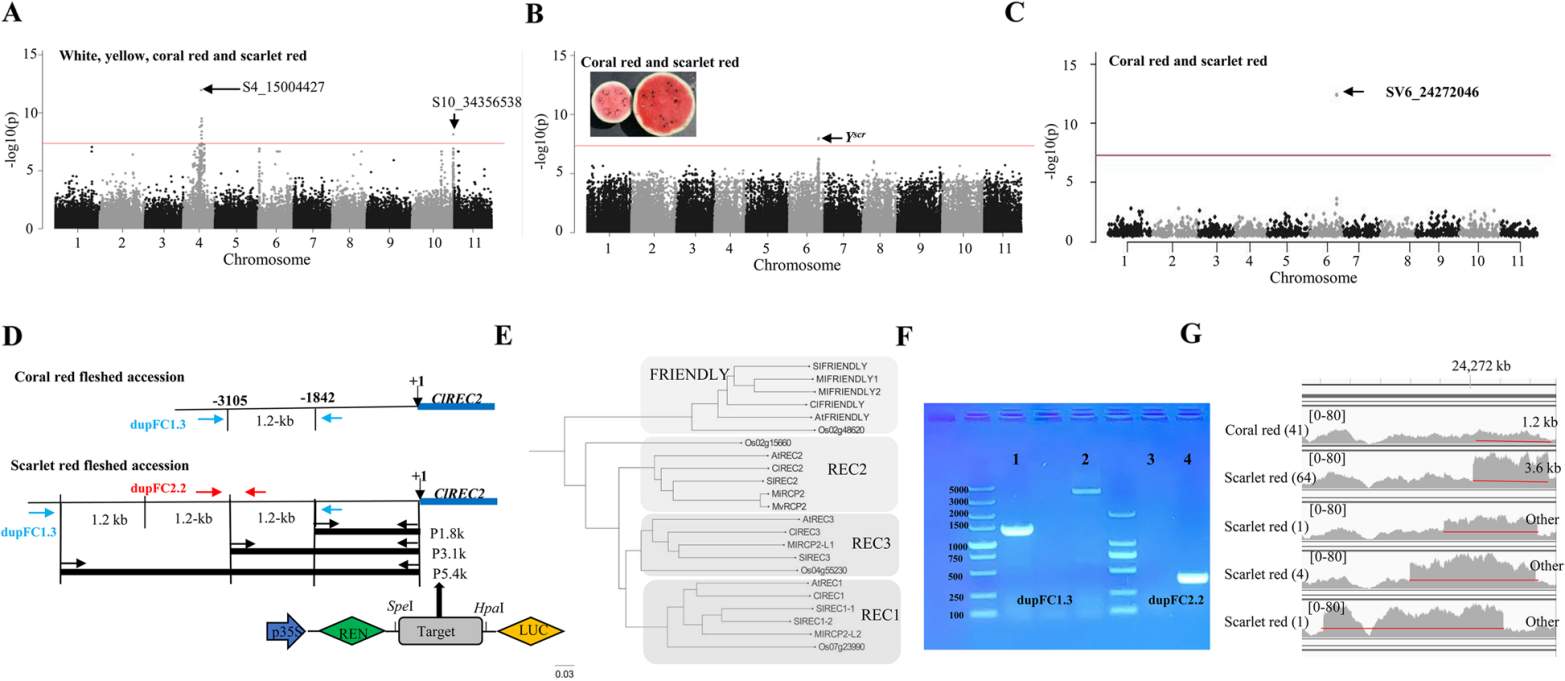
The CNV (SV6_24272046) was associated with flesh color in watermelon. **A**-**B** SNP-GWAS for flesh color. **C** SV-GWAS for flesh color. The horizontal red lines in A-C indicate the genome-wide threshold of GWAS signals. **D** CNV variation in the promoter of *ClREC2* between coral red and scarlet red fleshed watermelons. Three duplications of 1264 bp were found in scarlet red fleshed accessions. The P1.8k, P3.1k and P5.4k DNA fragments were PCR amplified and used as promoter target sequences to drive the expression of reporter gene *LUC*. Black, blue and red arrows represent positions of PCR-primers used to amplify target promoter target sequences and two markers dupFC1.3 and dupFC2.2. **E** Neighbor-joining phylogenetic tree of tetratricopeptide repeat proteins. The *Arabidopsis* sequences were retrieved from TAIR (www.arabidopsis.org). All other sequences were retrieved from Phytozome v13 (https://phytozome-next.jgi.doe.gov/). The tree was rooted by midpoint rooting. **F** PCR products were detected using primers for dupFC1.3 and dupFC2.2 and template DNA of a coral red flesh accession (lane 1,3) and scarlet red flesh watermelon (lane 2, 4). **G** A snapshot of IGV shows DNA sequence reads of 1 coral red and 4 scarlet red fleshed watermelons mapped to DR117 reference genome. It shows five patterns, each representing for 41, 64, 1, 4, and 1 watermelon accession(s) as indicated in the brackets. [0–80] shows the coverage of sequence depth

For fruit flesh colors, SNP-GWAS identified three strong peak clusters on chromosomes 4, 6 and 10 (Fig. 2A, B), respectively. The most strongly associated SNP was S4_15004427 (Fig. 2A) in *ClLCYB* showing a nonsynonymous G-T variation that converted phenylalanine (F) to valine (V) at the 226th amino acid position; which was the same as previously reported (Guo et al. 2019). A second signal associated with flesh color was found on chromosome 10, which is near the novel chromoplast phosphate transporter *ClPHT4;2* (Zhang et al. 2017) and is required for flesh color development in watermelon. A third signal separating coral red from scarlet red accessions was found on chromosome 6 (Fig. 2B) within the flesh color QTL *Y*^*scr*^ (Li et al. 2020). Furthermore, we identified an SV (SV6_24272046) was within the QTL *Y*^*scr*^ (Fig. 2C). A comparison of the reference genome sequences of G42 (scarlet red flesh) and DR117 (coral red flesh) revealed that SV6_24272046 was a CNV, a triplicate of a 1.2 kb segment spanning from –1842 bp to –3105 bp in the promoter region of *Chr6.g13111* gene in G42 (Fig. 2D). *Chr6.g13111* encodes a tetratricopeptide repeat protein (TRP) protein of 1,920 amino acids and contains a TPR domain with three TPR repeats, a CLUstered mitochondria protein N-terminal domain, and a CLU central domain. Phylogenetic analysis indicated Chr6.g13111 was closely related to the *Arabidopsis* REDUCED CHLOROPLAST COVERAGE 2 protein (REC2) (Fig. 2E). Hereafter, Chr6.g13111 is designated ClREC2. To confirm the presence of SV6_24272046 in different watermelon accessions, two PCR-based markers, dupFC1.3 and dupFC2.2, were developed based on the CNV, and used to genotype 41 coral red fleshed and 70 scarlet red fleshed watermelon accessions using PCR analyses (Fig. 2F, Table S12). It was found that all 41 coral red accessions had no triplication of the 1.2 kb, while 64 of the 70 scarlet red accessions had the triplication genotypes (Table S13). The other 6 scarlet red accessions had other types of duplications as showed by IGV analysis (Fig. 2G). These results confirmed the CNV was presence in all scarlet red watermelon accessions but not in coral red accessions, indicating an important role for this CNV in regulating flesh color.

To understand the domestication history of fruit flesh color, we further analyzed the allele distribution of these two variations in watermelon collections. We found one *C. colocynthis*, PI 195927, which exhibited segregation of flesh color (white and coral red flesh) (Figure S6A). Coral red fleshed PI 195927 had S4_15004427^GG (coral−red−allele)^, while white fleshed PI 195927 had S4_15004427^TT(white−allele)^ (Figure S6B); thus, the coral red allele appeared earliest in *C. colocynthis*. We also found segregation of flesh color (white flesh and coral red flesh) in *C. mucosospermus* accessions, PI 249010 and PI 247398 (Figure S6C, D). We found that the earliest CNV mutation occurred in one *C. lanatus*, PI 502319 with scarlet red flesh and collected from the Republic of Cameroon in the southeastern region of Africa. These results suggested that coral red flesh and its allele appeared during speciation and domestication but were largely fixed during improvement, while scarlet red flesh and its allele appeared and were largely fixed during improvement.

### The CNV for flesh color enhances the expression of *ClREC2*

**Fig. 3:**
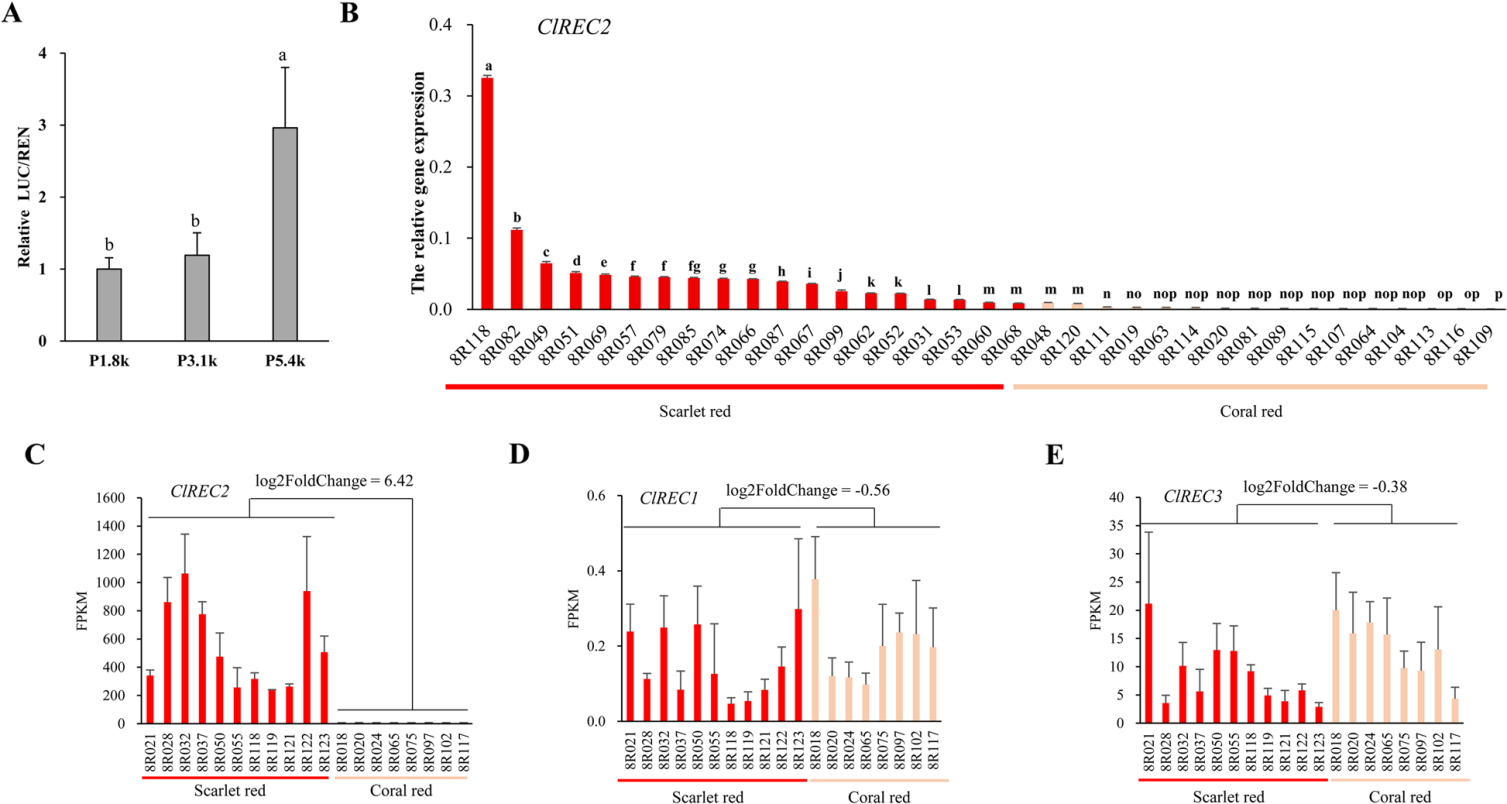
The CNV (SV6_24272046) regulated the expression of*ClREC2*. **A** Relative LUC/REN ratio was determined after watermelon protoplasts were transiently transformed with three constructs (35S:REN-P1.8k-LUC, 35S:REN-P3.1k-LUC and 35S:REN-P5.4k-LUC). The LUC/REN is the average ratio of the bioluminescence of firefly luciferase to that of Renilla luciferase. The values are the means ± SDs, with *n* = 3. Statistically significant differences were determined by one-way ANOVA; the different lower letters indicate significant differences according to Duncan’s test (*P* < 0.05). **B** The relative expression levels of *ClREC2* in mature flesh of 19 and 16 accessions as determined by qRT-PCR. Scarlet red fleshed 8R118 and coral red fleshed 8R020 was also used in RNA-seq analysis. The values are the means ± SDs (*n* = 3 biological replicates). Statistically significant differences were determined by one-way ANOVA; the different lower letters indicate significant differences according to Duncan’s test (*P* < 0.05). **C-E** FPKM value of *ClREC2*, *ClREC1* and *ClREC3* in 11 scarlet red and 8 coral red fleshed watermelon accessions. The values are the means ± SDs, with *n* = 3

As several putative cis-acting regulatory elements were identified in the 1.2 kb segment (Figure S7), we speculated that CNV (SV6_24272046) might directly regulates the expression of the downstream gene. A dual-luciferase reporter assay was performed to analyze the activity of promotors with different numbers of the 1.2 kb units. The two promoter fragments, 1.8 and 3.1 kb were PCR amplified from coral red accession 8R003 and contained none and one 1.2 kb unit, respectively. All primer sequences are listed in Table S12. The longest promotor of 5.4 kb was PCR amplified from scarlet red accession 8R001 and contained three 1.2 kb units (Fig. 2D). The 5.4 kb PCR amplicon was sequenced by PacBio Sequel II (Table S14) and confirmed to contain the triplicated unit (Fig. 2D). The dual-luciferase reporter assay showed that the promoter containing the triplicate had significantly higher activity than other two promoters (Fig. 3A), which suggested the triplicate CNV could enhance the expression of the downstream gene.

The enhancement of *ClREC2* expression by the triplication CNV was further confirmed by quantitative reverse transcription PCR (qRT-PCR) and transcriptome analyses. The relative expression of *ClREC2* was analyzed using qRT-PCR in 19 scarlet red (with the triplication) and 16 coral red (without the triplication) accessions. With exception of two coral red accessions (8R048 and 8R120), the *ClREC2* expression levels were significantly higher in scarlet red accessions with the triplication compared to coral red accessions without the triplication (Fig. 3B). Transcriptome analyses revealed that the transcript levels of *ClREC2* in 11 scarlet red accessions with the triplication were significantly higher than those in 8 coral red accessions without the triplication (Fig. 3C, log2foldchange = 6.42, p = 3.6E-305). *ClREC2* has two closely related homologous in watermelon, *ClREC1* and *ClREC3* (Fig. 2E). However, *ClREC1* and *ClREC3* exhibited a much lower level of expression (0.1 – 21 fragments per kilobase of transcript per million mapped reads (FPKM)) than *ClREC2* (240 – 1000 FPKM) and did not show differential expression between coral red and scarlet red accessions (Fig. 3D, E). Although ClREC1, ClREC2, ClREC3 may have similar function, approximate 100-fold increase of *ClREC2* expression caused by the triplication can mask any effect of *ClREC1* and *ClREC3*, and can confer a new phenotype.

### Chromoplast development and carotenoid contents are strongly associated with flesh color

**Fig. 4:**
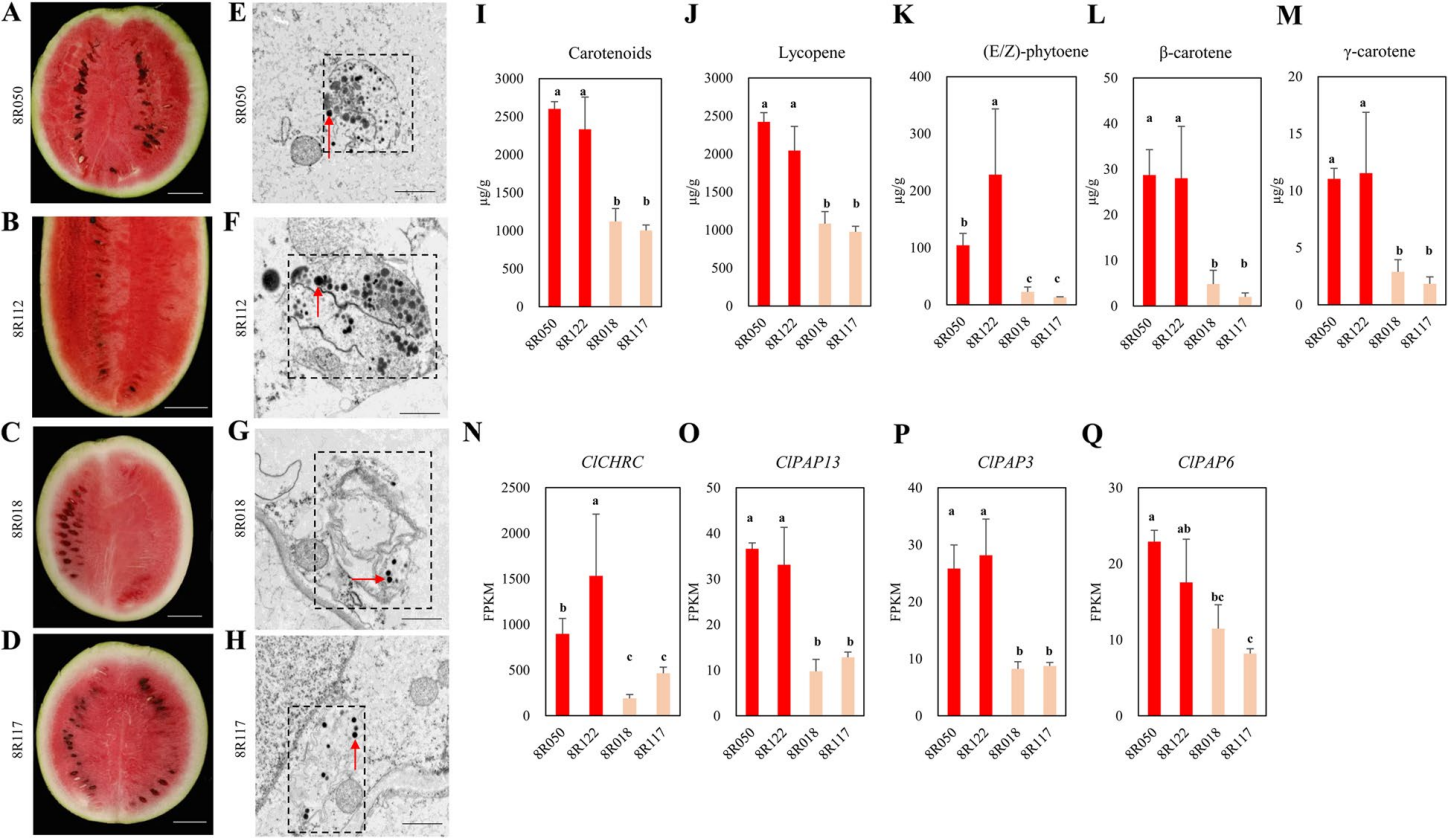
Comparison of chromoplast ultrastructure, carotenoid contents and expression level of chromoplast genes in different watermelons. **A-D** Photographs show longitudinal cross-sections of two scarlet red (8R050, 8R112) and two coral red (8R018, 8R117) accessions. Bars = 5 cm for A-D. **E–H** Electron microscopy image of chromoplasts in the flesh cells. The box covers one chromoplast and the red arrow points to a plastoglobulus within the chromoplast. Bar = 1 µm for E–H. **I-M** Content of total carotenoids, lycopene, (E/Z)-phytoene, β-carotene and γ-carotene in two scarlet red and two coral red accessions. **N-Q** The FPKM value of *ClCHRC*, *ClPAP13*, *ClPAP3* and *ClPAP6* in two scarlet red and two coral red accessions. The values are the means ± SDs, with n = 3 (I-Q). Statistically significant differences were determined by one-way ANOVA; the different lower letters indicate significant differences according to Duncan’s test (*P* < 0.05)

Watermelon flesh color is caused by carotenoids accumulated in chromoplasts of the flesh. Ultrastructure analysis of chromoplasts in flesh cells of mature fruits showed obviously visible carotenoid crystals and plastoglobulus in both scarlet red and coral red fleshed accessions (Fig. 4A-H). Plastoglobulus are carriers of carotenoid synthesis and accumulation (Bréhélin and Kessler 2008). Notably, more plastoglobulus were observed in the cells of two scarlet red fleshed watermelons than in two coral red fleshed genotypes (Fig. 4A-H). Thus, we further examined the carotenoid contents of four these watermelon accessions. Two scarlet red accessions accumulated significantly higher level of total carotenoids than two coral red accessions (Fig. 4I). The scarlet red fleshed accessions had significantly higher level of lycopene, (E/Z)-phytoene, β-carotene and γ-carotene than coral red fleshed accessions (Fig. 4J-M). In both type of accessions, most carotenoids are lycopene (Fig. 4J). Consistent with the increases plastoglobulus number in scarlet red fleshed watermelons, the expression level of genes encoding chromoplast proteins, ClREC2 (Fig. 3B, C), and ClCHRC (Fig. 4N), and encoding plastid lipid-associated proteins (ClPAP13, ClPAP3 and ClPAP6) (Fig. 4O-Q) were significantly higher in scarlet red fleshed accessions than in coral red accessions. These results indicated that enhanced expression of *ClREC2* and gene encoding other chromoplast proteins is positively correlated with the number of chromoplasts and carotenoid accumulation in fruit flesh cells.

### The expression of the MEP and CB genes are strongly associated with flesh color

**Fig. 5:**
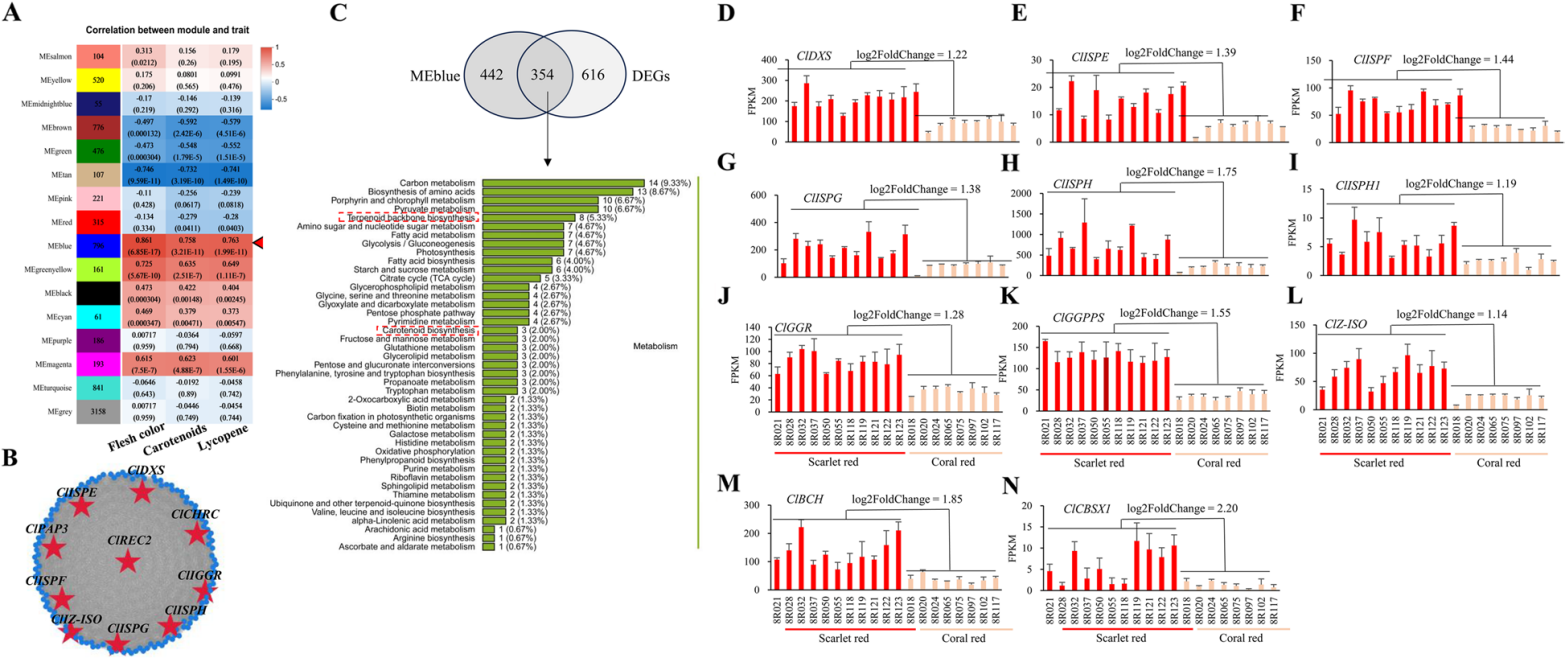
Hub genes related to flesh color in watermelon. **A** Heatmap of the correlation coefficient between WGCNA model eigengenes and flesh color, carotenoids and lycopene. The blue module MEblue was the most positively correlated with these traits. **B** Co-expression network of MEblue Showing the hub gene *ClREC2* connection to MEP and CB pathway genes. **C** KEGG pathway of 354 DEGs in MEblue. Terpenoid backbone biosynthesis and carotenoid biosynthesis were in red dashed box. **D-N** The transcript level of *ClDXS*, *ClISPE*, *ClISPF*, *ClISPG*, *ClISPH*, *ClISPH1*, *ClGGR, ClGGPPS*, *ClZ-ISO*, *ClBCH* and *ClCBSX1* in 11 scarlet red and 8 coral red fleshed watermelon accessions. The values are the means ± SDs, with *n* = 3

To further investigate the molecular mechanisms underlying the difference between scarlet red and coral red fleshed watermelons, we conducted comparative transcriptomic (Table S15) and metabolomic analyses. The expression matrix of 57 samples (11 scarlet red and 8 coral red accessions, and each with three biological duplicates) and 8247 expressed genes were used to construct a hierarchical clustering tree (Figure S8A) and a total of 16 co-expression modules were found (Figure S8B). Weighted gene coexpression network analysis (WGCNA) revealed that the blue module of the 16 co-expression modules showed the highest correlation with flesh color, carotenoid content and lycopene content, with Pearson's correlation coefficient of 0.861 (*p* = 6.85E-17), 0.758 (*p* = 3.21E-11) and 0.763 (*p* = 1.99E-11), respectively (Fig. 5A), implying the potential importance of the genes in the blue module in controlling flesh color and carotenoid accumulation. In the blue module, *ClREC2* was a key hub gene connecting to the MEP and CB pathway genes (Fig. 5B). The blue model contained 796 genes, which included *ClREC2*, chromoplast genes (*ClCHRC*, *ClPAP13*, *ClPAP3* and *ClPAP6*) and almost MEP and CB genes, including 1-deoxy-D-xylulose-5-phosphate synthase 2 (*ClDXS*), *ClISPD*, 4-diphosphocytidyl-2-C-methyl-D-erythritol kinase (*ClISPE*), 2-C-methyl-D-erythritol 2,4-cyclodiphosphate synthase (*ClISPF*), *ClISPG*, *ClISPH*, *ClGGPPS*, *ClPDS*, z-carotene isomerase (*ClZ-ISO*), z-carotene desaturase (*ClZDS*), carotenoid isomerase (*ClCRTISO*) and Beta-carotene 3-hydroxylase (*ClBCH*) (Table S16).

Comparative transcriptome analysis of 11 scarlet red and 8 coral red fleshed accessions identified a total of 970 differentially expressed genes (DEGs), 824 of which were upregulated in the scarlet red fleshed accessions. Of the 824 upregulated genes, 354 were in the blue coexpression module (Fig. 5C, Table S16), including 8 and 3 genes involved terpenoid backbone biosynthesis and carotenoid biosynthesis (Fig. 5C), respectively. DEGs in the blue coexpression module included *ClREC2* (Fig. 3C), *ClCHRC*, *ClPAP13*, *ClPAP3*, *ClPAP6* (Fig. 4N-Q), *ClDXS*, *ClISPE*, *ClISPF*, *ClISPG*, *ClISPH1*, *ClISPH*, geranylgeranyl reductase (*ClGGR*), *ClGGPPS*, *ClZ-ISO*, *ClBCH* and CBS domain-containing protein (*ClCBSX1*) (Fig. 5D-N). The log2fold changes of expression level for other 6 MEP and CB pathway genes (1-deoxy-D-xylulose 5-phosphate reductoisomerase (*ClDXR/ClISPC*), *ClISPD*, *ClPDS*, *ClIDI2*, *ClZDS* and *ClCRTISO*) were 0.60, 0.51, 0.86, 0.67, 0.96 and 0.90, respectively (Figure S9), which were lower than the threshold for defining DEGs. Moreover, of the 354 DEGs in the MEblue hub, 218 DEGs had been classified with function in chloroplast and chromoplast development (Table S16), which supports the conclusion that plastids play an important role in watermelon flesh color formation.

To validate *ClREC2* function, watermelon plants were infiltrated with the pV190-*ClREC2* virus-induced gene silencing (VIGS) construct to silence *ClREC2*, while plants infiltrated with the pV190 construct served as controls. *ClREC2*-silenced plants exhibited a chlorotic phenotype compared to the control plants (Figure S10A). Additionally, these plants showed reduced expression levels of chlorophyll-related genes (Figure S10B), but maintained normal chloroplast ultrastructure and chlorophyll content (Figure S10C, D). Notably, the expression levels of not only *ClREC2*, but also MEP and CB pathway genes were significantly reduced in pV190-*ClREC2* plants compared to pV190 plants (Figure S10E). These results further confirmed that *ClREC2* functions as a positive regulator of carotenoid biosynthesis.

### A “two-switch” model regulating flesh color in watermelon

**Fig. 6:**
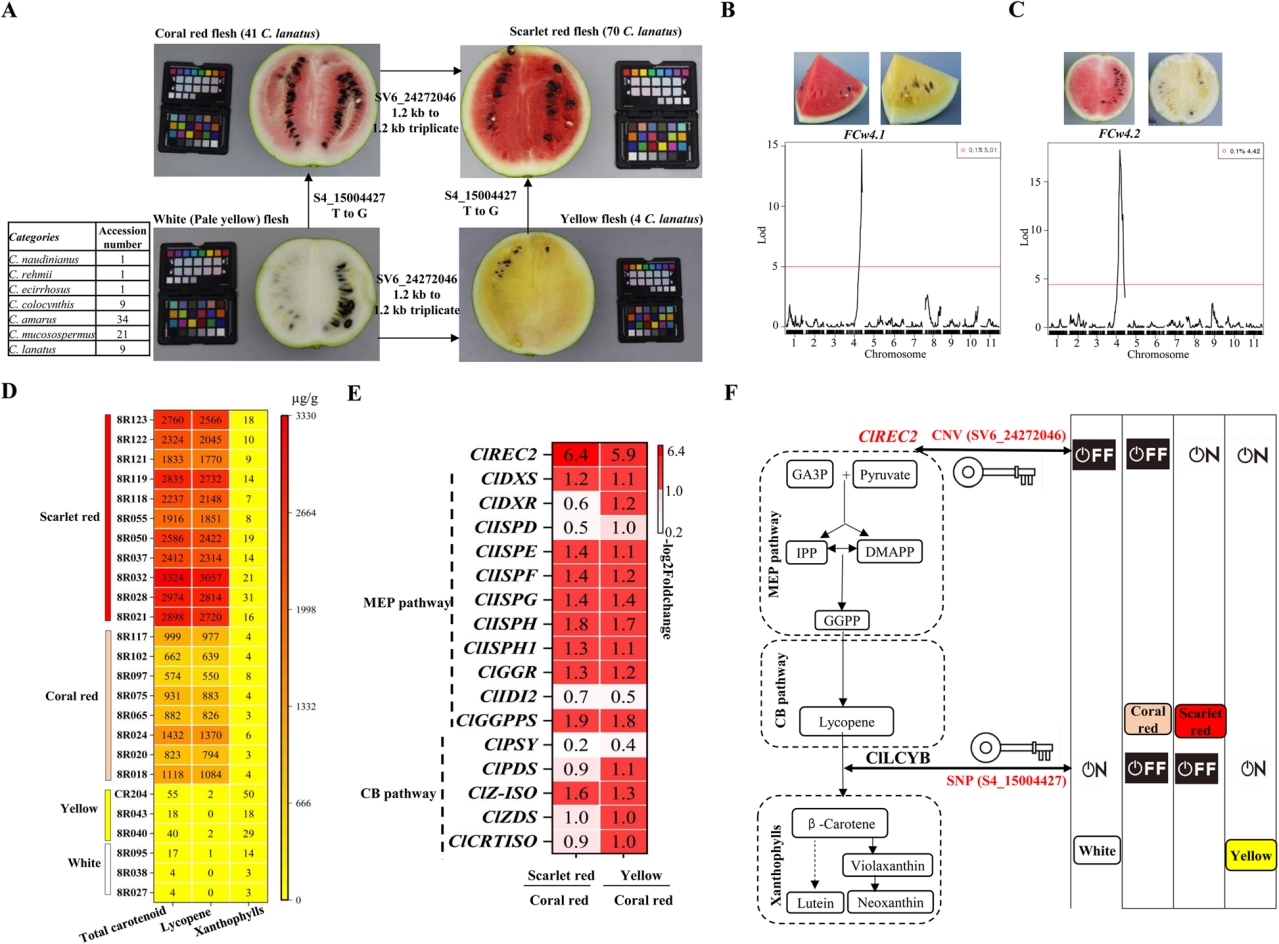
Two genetic variants together regulate the formation of four flesh colors of watermelon. **A** The genetic model for four flesh colors in watermelon. **B** QTL mapping using a BC_1_F_1_ population derived from a cross between a scarlet red and a yellow fleshed accession. **C** QTL mapping using a BC_1_F_1_ population derived from a cross between a coral red and a white accession. **D** Heatmap of carotenoid content in 25 watermelon accessions with four different flesh colors. **E** The heatmap of genes in MEP and CB pathways. The number in heatmap was the log2FoldChange in FPKM between scarlet red and coral red, between yellow and coral red watermelon accessions.** F** A proposed two-switch model explaining the regulation of watermelon flesh color. The dashed box shows the simplified of MEP, CB and xanthophyll pathways, respectively. Two variants SV6_24272046 and S4_15004427 act as switches to turn on or off *ClREC2* and *ClLCYB*, respectively. The combinations of the on and off of the two genes determine the four colors (white, coral red, scarlet red and yellow) of watermelon fruit flesh

Our analysis of the 196 watermelon core accessions began to reveal an integrated genetic model of the SV6_24272046 and S4_15004427 variants for regulation of flesh color. The analysis showed that 76 white fleshed (9 *C. lanatus* and one *C. naudinianus*, one *C. rehmii*, one *C. ecirrhosus*, 9 *C. colocynthis*, 34 *C. amarus* and 21 *C. mucosospermus*) had the wild-type allele for both variants (Fig. 6A). All 41 coral red accessions of *C. lanatus* had mutant allele of S4_15004427 containing a point mutation in *ClLCYB*. This indicated that the point mutation in *ClLCYB* may convert white flesh to coral red flesh. In addition, 4 yellow fleshed *C. lanatus* accession had the mutant allele of SV6_24272046 containing the 1.2 kb triplicate, indicating that the triplication could convert white flesh to yellow flesh. Finally, all 70 scarlet red accessions of *C. lanatus* had the mutant allele of both SV6_24272046 and S4_15004427 (Fig. 6A), indicating both mutations together could enhance red color development.

To support the above genetic model, we further analyzed the flesh color and genotype of 314 accessions with different fruit flesh colors (Table S13). This analysis showed that these two variants explained 99.7% of the phenotypic variation in all four flesh-colored groups of accessions. Only one accession, the Chinese landrace 8R095 (Daoxianhongzigua) had white flesh color but showed the 1.2 kb triplication. The 1.2 kb triplicate in 8R095 was confirmed by IGV and PCR analysis with the dupFC2.2 marker (Table S13).

Further support of the genetic model came from analyses of two BC_1_F_1_ populations. First, analysis of a BC_1_F_1_ (19QB12) population derived from the cross between a scarlet red fleshed accession (B85, Xiaoxigua) and a yellow fleshed line (B10, CHHX) detected a significant QTL (*FCw4.1*; LOD score = 19.6; R^2^ = 82.76%) on chromosome 4 (Fig. 6B), which co-located with S4_15004427. This result indicated that mutation of *ClLCYB* can convert yellow flesh to scarlet red flesh (Fig. 6A). Secondly, analysis of another BC_1_F_1_ (19QB9) derived from a cross between a coral red fleshed accession (J) and a white fleshed accession (CreamSuika) also detected a significant QTL (*FCw4.2*; LOD score = 20.3; R^2^ = 94.71%) on chromosome 4 (Fig. 6C), co-located with S4_15004427 and *FCw4.1*. This result indicated that mutation of *ClLCYB* can convert white flesh to coral red flesh (Fig. 6A).

Different fruit flesh colors of watermelon is determined by the different carotenoids accumulated in the flesh, so we analyzed the carotenoid composition and contents of 3 white, 3 yellow, 8 coral red and 11 scarlet red watermelon accessions. The result (Fig. 6D) showed that the three white accessions accumulated a very low level of total carotenoids (4—17 µg/g), mainly the yellow pigment xanthophylls. The three yellow accessions accumulated a slightly higher level of carotenoids (18—55 µg/g), again mainly xanthophylls. In contract, the eight coral red accessions accumulated a high level of carotenoids (574 – 1432 µg/g), mainly the red pigment lycopene. The 11 scarlet red accessions accumulated even higher levels of carotenoids (1833 – 3324 µg/g) compared to the coral red accessions, again mainly lycopene (Fig. 6D). These results indicate that the four main flesh colors of watermelon, white, yellow, coral red and scarlet red, are correspond to a very low level of any carotenoid, low level of xanthophylls, high level of lycopene, and even higher level of lycopene, respectively.

By integrating the results of genomic, transcriptomic, and metabolic analyses, we developed a “two-switch” model for explaining the regulation of flesh color in watermelon. In the model, the transcript level of *ClREC2* and all MEP and CB pathway genes are upregulated in scarlet red accessions compared to coral red accessions, and in yellow accessions compared to coral red accessions (Fig. 6E). This indicates that SV6_24272046 mutant allele containing the 1.2 kb triplicates in the promoter of *ClREC2* enhances the expression of *ClREC2* and subsequently MEP and CB pathway genes, like “switch on”. When *ClREC2* is on, watermelon accessions have SNP (S4_15004427) mutant allele blocking *ClLCYB* function, like “switch off”, their flesh accumulates a very high level of lycopene, thus showing scarlet red color. If accessions have SNP (S4_15004427) WT allele maintaining ClLCYB function, like “switch on”, their flesh accumulates xanthophylls, thus showing yellow color. When both switches are on, the yellow accessions are expected to accumulate a good level of xanthophylls. However, the yellow accessions accumulated a relative low level of xanthophylls (Fig. 6D). This may be explained by a high instability of these compounds in watermelon, which needs to be analyzed in future studies.

The model (Fig. 6F) further predicts that SV6_24272046 wild type allele does not enhance the expression of *ClREC2* or the pathway genes, like “switch off”. When ClREC2 is off, watermelon accessions also have SNP (S4_15004427) at “switch off”, the accessions accumulate a good level of lycopene, thus showing coral red flesh. If SNP (S4_15004427) is at “switch on”, the accessions accumulate little β-carotene or xanthophylls, thus showing white flesh.

## Discussion

A high-quality reference genome of watermelon, DR117, was reported and used to develop integrated genetic maps with SNP and SV markers in this study. A total of 11 superscaffolds corresponding to the 11 chromosomes were assembled, with scaffold N50 = 35.2 Mb. The contiguity and the number of predicted genes for DR117 reference genome are comparable to G42 (Deng et al. 2022), and higher than other six assembled reference genomes (Guo et al. 2019; Wu et al. 2023; Wu et al. 2019; Renner et al. 2021). High-quality reference genomes are the basis for understanding genome structure, genetic variation, evolution and domestication of an organism. Using the DR117 reference together with SNP and SV markers, we identified a major QTL on chromosome 6 controlling watermelon flesh color. The QTL is the same as that reported previously (Li et al. 2020; Yi et al. 2023). However, the novelty of this study is that we identified a CNV, SV6_24272046 marker in *ClREC2* promotor within the QTL and further showed that the CNV is a triplicate of 1.2 kb DNA fragment by comparing the reference genome sequence of DR117 (coral red flesh) and G42 (scarlet red flesh). The CNV could enhance the expression of *ClREC2* (Fig. 3B, C) because of increasing the number of cis-acting regulatory elements (Figure S7), sharing a similar mechanism reported for the apple *MdMYB10* allele containing 6 repeats of a minisatellite in the promotor (Espley et al. 2009).

The CNV (SV6_24272046) variant of *ClREC2* explained 100% of the phenotypic variation in 70 scarlet red and 41 coral red fleshed watermelon accessions. The CNV with triplication significantly enhanced the expression of the downstream reporter gene compared to that without triplication in a dual-luciferase reporter assay (Fig. 3A). Furthermore, transcript analysis revealed that *ClREC2* expression levels were significantly higher in 29 scarlet red accessions with the triplication than in 23 coral red accessions without the triplication (Fig. 3B, C). These results strongly suggested that the CNV in *ClREC2* promoter directly infulences *ClREC2* expression level, thereby underlies flesh color variation in watermelon.

ClREC2 is a member of the a tetratricopeptide repeat protein family, cluster together with REC2 protein (Larkin et al. 2016) and REDUCED CAROTENOID PIGMENTATION 2 (RCP2) (Stanley et al. 2020) through phylogenetic analysis (Fig. 2E). Loss of function *REC* mutants in *Arabidopsis* have reduced chlorophyll contents and smaller chloroplast compartment size (Larkin et al. 2016). Loss of function mutants in *RCP2* in monkeyflowers (*Mimulus*) cause drastic down-regulation of the all genes in the CB pathway, thus reduces carotenoid biosynthesis (Stanley et al. 2020). In the *rcp2* mutant of monkeyflowers, the chromoplast are skinny, irrgularly shaped and contain few plastoglobuli (Stanley et al. 2020). Repression of *RCP2* translation and transcription by siRNAs produced from the *YELLOW UPPER* locus ultimately led to the lack of yellow pigment accumation in the petals of *Mimulus lewisiii* (Liang et al. 2023). These observations suggested that *REC* and *RCP2* are master regulators of pigmentation through control plastid development. The present study showed that the triplicate of CNV (SV6_24272046) contained in scarlet red watermelons could enhance the expression of *ClREC2* and genes in both MEP and CB pathways. These two pathways produce precursors or products of carotenoid biosynthesis. As a consequence, scarlet red fleshed watermelons produced a significantly higher level of carotenoids than coral red watermelons without the triplicate CNV (Fig. 6D). The expression levle of MEP and CB genes were significantly lower in *ClREC2*-silenced plants compared to control plants (Figure S10D). The previous study showed that knockout of RCP2 reduced carotenoid accumilation (Stanley et al. 2020) while our study showed that activation of ClREC2 increased carotenoid accumilation. Our findings support the role of RCP2 and REC poteins in regulation of carotenoid accmuliation and further show that ClREC2 plays a core role in the formation of different flesh colors in watermelons.

Chromoplasts and chloroplasts are known to be interconvertible, chromoplasts are special organelles being the location for the synthesis and accumulation of carotenoid pigments in many colored flowers, fruits, and vegetables (Li and Yuan 2013). Plastoglobules is a lipoprotein particle within chromoplasts and is suggested to be involved in chromoplast biogenesis, and carotenoid synthesize, sequestration and storage (Bréhélin and Kessler 2008; Ytterberg et al. 2006). Our results showed that the number of plastoglobules per chromoplast was higher in scarlet red watermelons than in coral red watermelons (Fig. 4A-H). The plastoglobule number is positively correlated with the expression level of genes encoding chromoplast proteins and the content of catotenoids in watermelon flesh, thus flesh color. Our results suggested that *ClREC2* upregulation might affect plastoglobules formation as well as carotenoid accumulation.

In addition to the CNV (SV6_24272046), a SNP variant (S4_15004427) in the coding sequence of *ClLCYB* is also critial to watermelon flesh color variation. Previous studies have suggested that a decrease in the abundance of the ClLCYB protein caused by the SNP on chromosome 4 contributes to red flesh color in domesticated watermelon (Guo et al. 2019; Zhang et al. 2020). The same locus was also detected by using comparisons between white and red, and between pale or canary yellow and red (Zhang et al. 2020; Branham et al. 2017). The present study detected the same SNP on chromosome 4 (S4_15004427) by using comparison between scarlet red and yellow, and between white and coral red watermelon accessions (Fig. 6B, C). The present study further confirmed the strong association between these two major markers (SV6_24272046, and S4_15004427) and flesh color by analyzing 314 watermelon accessions with different flesh colors (Table S13). Our study, along with previous research (Zhang et al. 2020), revealed that the white, orange and yellow fleshed accessions carry the S4_15004427^TT^ alleles (corresponding to “ClLCYB” off), while pink (coral red) and red (scarlet red) fleshed accessions possessed the S4_15004427^GG^ alleles (corresponding to “ClLCYB” on). Downregulation of *ClLCYB* through genetic transformation of watermelon changed the flesh color from pale yellow to red, whereas overexpression of *ClLCYB* in the red fleshed accessions resulted in an orange flesh color (Zhang et al. 2020). These findings collectively establish ClLCYB as the molecular switch governing the lycopene/β-carotene branching point in watermelon carotenogenesis.

Integrating the results of genomic, transcriptomic, and metabolic analyses of this study together with previous reported ClLCYB and REC2 function (Zhang et al. 2020; Larkin et al. 2016; Stanley et al. 2020), we proposed a two-switch regulatory model to explain how the two variants (SV6_24272046 and S4_15004427) control watermelon flesh color together (Fig. 6F). The model suggestes that scarlet red fruit flesh is resulted from switching on the CNV marker to increase the biosynthesis of lycopene and switching off the SNP marker to block the convertion of lycopene to β-carotene, thus accumulation of a very high level of the red piaments lycopene. When both marker are on, a yellow flesh is produced due to convertion of lycopene to the yellow pigment β-carotene. When both switchers are off, a coral red flesh is produced due to a ccumulation of a medium level of lycopene. When the CNV switcher is off, but the SNP switcher is on, a white flesh is produced due to little accumulation of any pigment.

Lycopene is the predominant carotenoid in both coral red and scarlet red watermelon varieties (60%-78% of total carotenoids), whereas yellow-fleshed fruits accumulated primarily violaxanthin (40–45%). At 10 days after pollination (DAP), fruit flesh of all varieties is unpigmented with white flesh containing no detectable carotenoids according to Yuan et al. (2021). Based on reanalyzing the data of Yuan et al., lycopene content significantly increases from 20 to 32 DAP in coral red (2.37-fold) and scarlet red (2.34-fold) varieties, with scarlet red accumulating 42% more lycopene than coral red (Figure S11A). Yellow-fleshed fruits showed a 1.35-fold increase in violaxanthin accumulation from 20 to 32 DAP (Figure S11B). Intriguingly, *ClREC2* expression displayed cultivar-specific regulation rather than being strictly developmental-stage dependent. Within each flesh color group, *ClREC2* transcript levels remained stable across 10, 20, and 32 DAP (Figure S11C). However, *ClREC2* expression was significantly higher in scarlet red and yellow varieties (CNV switcher “on”) compared to white and coral red cultivars (CNV switcher “off”). These findings suggests that *ClREC2* alone is not sufficient to initiate carotenoid biosynthesis, implying the involvement of additional regulatory factors beyond ClREC2-mediated control.

In conclusion, we report an assembly of high-quality watermelon reference genome, integrated genetic maps with SNP and SV markers, and the use of these resources to uncover key genetic variants and molecular mechanisms underlying flesh color and other fruit traits. We also propose a two-switch regulatory model elucidating the coordinated control of flesh color and carotenoid biosynthesis in watermelon flesh. Our findings not only provide novel insight into the molecular mechanisms underlying the historical domestication and improvement of watermelon fruit characteristics, but also establish a robust genomic foundation for targeted manipulation of carotenoid synthesis and nutritional quality in plants. The developed resources and mechanistic model offer valuable tools for both fundamental plant biology research and precision breeding applications.

## Methods

### Plant materials and sequencing

The diploid watermelon inbred line DR117 showing coral red flesh was selected for assembling a reference genome. From young fresh leaves, high-molecular-weight DNA was isolated and used to make a 20 kb library that was sequenced on the Pacific Biosciences Sequel platform. For optical mapping, high-molecular-weight DNA was isolated from young yellow leaves after dark treatment and labeled with the DLE-1 enzyme (BioNano Genomics). The labeled DNAs were scanned with the Saphyr system. For PacBio Iso-Seq, a total of 11 samples of sprout, leaf, ovary, seed, and fresh fruit tissue at 1 and 2 days after flowering and fruit flesh and rind at 15, 20 and 30 DAP were collected. The Iso-Seq libraries were constructed following the standard SMRT bell construction protocol and sequenced on 2 SMRT cells (for 1–10 kb and 4–10 kb libraries) using the Sequel platform.

A total of 196 watermelon accessions from the National Mid-term Genebank for Watermelon and Melon (Zhengzhou, China) with diverse genetic backgrounds were used for resequencing (Table S5). Genomic DNA was extracted from fresh young leaves using a Plant Genomic DNA Kit (Tiangen). Libraries with 350 bp inserts was prepared and sequenced on an Illumina NovaSeq6000 platform for 150 bp paired-end reads. In addition, 175 watermelon accessions, which had been sequenced previously (Guo et al. 2019), were used to confirm the key variations in important traits.

For transcriptome sequencing, total RNA (each with three biological replications) was extracted from flesh of mature fruit using the QIANEN RNase Plant Mini Kit (Qiagen). Strand-specific RNA-seq libraries were prepared and sequenced on an BGISEQ platform with 150 bp paired-end reads.

To locate the major QTLs for flesh color, two BC_1_F_1_ (19QB9, 19QB12) were used, of which the genomes of individuals were sequenced via restriction-site-associated DNA sequencing.

### Planting and phenotyping

In total, 196 watermelon accessions were grown in Zhengzhou (Henan Province), Changji (Xinjiang Province) and Sanya (Hainan Provence) in 2018 and 2019. Phenotyping of 129 *C. lanatus* accessions was performed at six locations, while the other accessions were evaluated in Zhengzhou in 2018. Three replicates with 10 plants each were planted at each location. We phenotyped watermelons for the qualitative traits of flesh color (white, coral red, scarlet red and yellow), fruit shape (elongated and spheroidal). Each fruit was cut lengthwise and measured at harvest. Fruit length (cm) and fruit width (cm) were measured using a ruler. The fruit shape index was calculated as the ratio of fruit length to fruit width. All lines were phenotyped following a Chinese specification for evaluating watermelon (Ma and Liu 2005).

### De novo assembly and gene annotation

De novo assembly was conducted with PacBio SMRT long reads and Canu software (Koren et al. 2017). Pilon2 (Walker et al. 2014) was used to perform the second round of error correction with Illumina short read data. BioNano optical maps labeled by DLE-1 were assembled into consensus physical maps with the Assembler tool in the BioNano Solve package. The Bionano Solve software imports the assembly and identifies putative nick sites in the sequence based on the nicking endonuclease-specific recognition site. These in silico maps for the sequence contigs are aligned to the de novo Bionano genome maps. Protein annotations were carried out by searching the NCBI nonredundant protein, InterPro, and KEGG databases. GO information was extracted from InterPro annotation (Zdobnov and Apweiler 2001; Quevillon et al. 2005). Jellyfish (Marcais and Kingsford 2011) was used to perform k-mer analysis on the Illumina sequencing error-corrected data.

### Core collection selection

The program Core Hunter 3 (Beukelaer et al. 2018) was used to identify a subset of accessions that captures the majority of the allelic diversity of the 1022 watermelons. SnpReady software (Granato et al. 2018) was used to assess the diversity captured in the core collections relative to the initial collection using multiple evaluation indices. The core collection was further evaluated by phylogenetic analysis.

### SNP and SV detection

Paired-end reads were mapped to DR117 using BWA (Li and Durbin 2009). SNP calling was performed using GATK HaplotypeCaller, and genotyping was performed with GenotypeGVCFs (DePristo et al. 2011). LUMPY (Layer et al. 2014) was used to detect SVs with the except of insertions. The SV results were then genotyped in the population using SVTyper (Chiang et al. 2015). SURVIVOR (Jeffares et al. 2017) was applied to define and merge SVs. A manual check by comparing 499 deletions and 55 duplications on chromosome 6 with the PacBio long read mapping results using the IGV program (Robinson et al. 2011) revealed an accuracy rate of 85.2%.

### Genetic diversity and selective sweep analysis

Population genetic analyses were performed based on representative SNPs. The whole genome was divided into 5,000 fragments of equal length. From each fragment, 15 SNPs were randomly selected. If the number of SNPs was less than 15 in a fragment, all SNPs were selected. The phylogenetic tree was generated with the neighbor-joining method. STRUCTURE (Falush et al. 2003) was used to infer population structure by using 10,000 iterations with K values ranging from 2 to 10. LD analyses for each subpopulation were performed using PopLDdecay (Zhang et al. 2019). Pairwise IBS calculations were performed using PLINK (Chang et al. 2015). θπ and F_ST_ values were calculated with VCFtools (Danecek et al. 2011). The average Fst value was calculated in each 1-Mb length window with 100-kb length steps and sliding windows with the top 5% of the Fst were selected as candidate highly divergent regions.

### GWAS and QTL mapping

To improve the accurate of the GWAS results, we filtered the SNP and SV datasets by removing those with minor allele frequency < 0.01. The association analysis for SNP was performed using the mixed linear model (MLM) method incorporated into TASSEL (Bradbury et al. 2007) software. The Q matrix was determined using STRUCTURE (Falush et al. 2003). The pairwise kinship coefficients were estimated in TASSEL software. The association analysis for SV was performed using MLM, GLM, CMLM and FarmCPU models in GAPIT (Lipka et al. 2012), which also integrates PCA and kinship analyses. A stringent Bonferroni correlation was used to screen obvious association signals based on P value (significant threshold: -log_10_(P) ≥ 7.35 for SNP and 6.13 for SV) divided by 0.05. Only the SNPs whose segregation patterns were aa × bb were used to construct the genetic map. The genetic map was constructed using MSTmap (Wu et al. 2008). QTL mapping was performed using the composite interval mapping in the R/qtl package (Arends et al. 2010).

### Development of PCR-based markers and genotyping

The primers for dupFC1.3 designed to span the entire CNV fragment and amplify based on elongation time to distinguish different flesh color. The primers for dupFC2.2 designed to span the CNV breakpoint could amplify the expected fragment in scarlet red watermelons, but could not in coral red watermelons. The genotyping of markers were performed as previously (Li et al. 2020). The elongation time for PCR amplification varied from the length of the products and usually amplified 1 kb per min. Products with lengths less than 1 kb were separated on an 8% polyacrylamide gel and visualized by silver staining. Products with lengths greater than 1 kb were separated on a 1% agarose gel and visualized by Goldview.

### Dual-luciferase reporter assays in protoplasts

The target sequences of P1.8k and P3.1k were amplified from coral red watermelon 8R003 and the target sequences of P5.4k were amplified from scarlet red watermelon 8R001. The target sequences were inserted into the PUC57-StrigoQuant-like backbone vector (Zheng et al. 2020) using an in-Fusion HD cloning kit through the use of SpeI and HpaI. The vectors of P1.8k and P3.1k were sequenced by Sanger sequencing. The vector of P5.4k was sequenced by PacBio Sequel II and assembled by smrtlink11.0.0. The vectors were used to transforme watermelon protoplasts as previously (Tian et al. 2017) with minor improvement. The Renilla (REN) and LUC activities were measured using a dual-luciferase reporter assay system (Promega).

### Quantification of gene expression and WGCNA

Transcriptome sequence was aligned to DR117 using HISAT2. Subread software was used to count the reads mapped to each gene, and FPKMs were calculated (Trapnell et al. 2010). Expressed genes were defined using a threshold of 0.5 in FPKM. The WGCNA was carried out utilizing the Majorbio Cloud Platform (https://cloud.majorbio.com/page/tools/). Differential expression analysis was performed using the DESeq R package (Love et al. 2014). DEGs between groups were determined by absolute fold change of fragments per kilobase million (FPKM) > 1 and pval ≤ 0.05.

### VIGS assay and qRT-PCR

We used VIGS technique with cucumber green mottle mosaic virus-based vectors (pV190) to silence the *ClREC2* gene, following the method described previously (Liu et al. 2020). qRT-PCR was performed as described previously (Li et al. 2020) using the watermelon actin gene as reference gene. The primer sequences were listed in Table S12. The chlorophyll and carotenoid content in leaf was measured according to methods described previously (Arnon 1949).

### Transmission electron microscopy

Watermelon fruit flesh or leaf was treated with a fixative and then washed with Pipes buffer (PB, PH = 7.4). Tissues were then post-fixed in 1% OsO4 in 0.1 M PB for 7 h at room temperature in dark. After three washes with PB for 15 min each, the fruit flesh or leaf was dehydrated in a concentration gradient of ethanol. The samples were penetrated in 3:1 acetone: EMBed 812 for 2–4 h, 1:1 acetone: EMBed 812 overnight, 1:3 acetone: EMBed 812 for 2–4 h, pure EMBed 812 for 5–8 h, and polymerized in pure EMBed at 60 °C for 48 h. Ultrathin sections were cut and stained sequentially in a 2% uranium acetate saturated alcohol solution and 2.6% lead citrate for 8 min each. Images of the samples were captured using a transmission electron microscope (HITACHI, Japan).

### Determination of carotenoid composition and concentrations

Carotenoid contents were detected based on the AB Sciex QTRAP6500 LC–MS/MS platform. Mature fruit flesh (each with three biological replications) was collected, freeze-dried and ground into fine powder. 50 mg powder was extracted with mixture of n-hexane:acetone:ethanol (1:1:1, V/V/V). After two extractions, the supernatant was evaporated to dryness under nitrogen, and reconstituted in 100 μL dichloromethane. The solution was filtered through a 0.22 μm membrane filter for further UPLC-APCI-MS/MS system. The analytical conditions were as follow, LC: column, YMC C30 (3 μm, 100 mm × 2.0 mm i.d); solvent system, methanol:acetonitrile (1:3, v/v) with 0.01% BHT and 0.1% formic acid (A), methyl tert-butyl ether with 0.01% BHT (B); gradient program, started at 0% B (0–3 min), increased to 70% B (3–5 min), then increased to 95% B (5–9 min), finally ramped back to 0% B (10–11 min); flow rate, 0.8 mL/min; temperature, 28°C; injection volume: 2 μL. Linear ion trap and triple quadrupole scans were acquired on a triple quadrupole-linear ion trap mass spectrometer (QTRAP), QTRAP® 6500 + LC–MS/MS System, equipped with an APCI Heated Nebulizer, operating in positive ion mode and controlled by Analyst 1.6.3 software. The APCI source operation parameters were as follows: ion source, APCI + ; source temperature 350°C; curtain gas was set at 25.0 psi. Carotenoids were analyzed using scheduled multiple reaction monitoring. Data acquisitions were performed using Analyst 1.6.3 software. Multiquant 3.0.3 software was used to quantify all metabolites. Mass spectrometer parameters including the decluttering potentials (DP) and collision energies (CE) for individual MRM transitions were done with further DP and CE optimization. A specific set of MRM transitions were monitored for each period according to the metabolites eluted within this period. The database was built based on authentic carotenoid standards for the qualitative analysis of MS data. Then, for absolute quantification, we prepared the solutions for each carotenoid standard with several different concentrations and obtained the peak area values corresponding to each concentration. Next, we separately constructed standard curves for all authentic carotenoid standards. Thereafter, we calculated the concentration values for all carotenoids using their respective standard curves. Finally, we determined the contents of the targeted carotenoids (µg/g) in different biological samples using the formula c*V/1000/m, where c represents the concentration value (µg/mL) calculated using the standard curve, V represents the reconstitution volume (µL), and m represents the sample weight (g). The carotenoid determination service was provided by Bioyi Biotechnology Co., Ltd. Wuhan, China.

## Supplementary Information

Supplementary Material 1: Figure S1. Eleven superscaffolds (chromosomes) of the DR117 genome corresponding to 11 chromosomes of the 97103v2 and G42 genomes.

Supplementary Material 2: Figure S2. K-mer analysis of the watermelon genome using GenomeScope.

Supplementary Material 3: Figure S3. Development and evaluation of the watermelon core collection. A, Coverage of allelic diversity versus number of selected accessions analyzed. B, Neighbor-joining phylogenetic trees of 1022 *Citrullus* accessions. The core collections are indicated by red dots.

Supplementary Material 4: Figure S4. Neighbor-joining phylogenetic trees of *Citrullus* accessions. Two* C. lanatus* accessions on the deepest branch of the *C. lanatus* clade and one *C. mucosospermus* accession on the lowest branch of the *C. mucosospermus* clade are indicated by the arrow.

Supplementary Material 5: Figure S5. SNP-GWAS and SV-GWAS identify loci of fruit shape in watermelon. A, SNP-GWAS for fruit shape. B, SV-GWAS for fruit shape. C, The location of causative variants in the candidate gene *ClSUN* related to fruit shape. The horizontal red lines in A-B indicate the genome-wide threshold of GWAS signals.

Supplementary Material 6: Figure S6. The phenotypes and genotypes of *C. colocynthis* accession and two C. *mucosospermus* accessions with separation of flesh color. A, The fruit and flesh of PI 195927. B, Enzyme digestion products after PCR amplification by primer 4FC1.5. C, The fruit and flesh of PI 249010. D, The fruit and flesh of PI 247398.

Supplementary Material 7: Figure S7. Nucleotide sequence of the 1263 bp with putative cis-acting regulatory elements shown. The important putative cis acting regulatory elements are underlined with red lines. The “TGACG-motif” is a cis-acting regulatory element involved in the MeJA-responsiveness. The “TCA-element” is a cis-acting element involved in salicylic acid responsiveness. The “MRE” is a MYB binding site involved in light responsiveness. The “ARE” is a cis-acting regulatory element essential for the anaerobic induction. The “G-box” is a cis-acting regulatory element involved in light responsiveness.

Supplementary Material 8: Figure S8. Cluster dendrogram of genes in watermelon. A, Cluster dendrogram of genes and corresponding modules. Each color represents a module, and the gray color represents genes not included in any module. B, Heatmap of the correlations between modules.

Supplementary Material 9: Figure S9. The gene expression of *ClDXR*, *ClISPD*, *ClIDI2*, *ClPDS*, *CZDS* and *ClCRTISO *in 11 scarlet red and 8 coral red fleshed watermelons. FPKM of *ClDXR *(A),* ClISPD* (B), *ClIDI2* (C), *ClPDS *(D), *ClZDS* (E),* ClCRTISO* (F) in 11 scarlet red and 8 coral red fleshed watermelon, respectively. The values are the means ± SDs, with n = 3.

Supplementary Material 10: Figure S10. Silencing* ClREC2* in watermelon using the VIGS vector pV190. A, Watermelon leaf was infiltrated with the empty vector pV190 served as a control and another leaf was infiltrated with the vector pV190-*ClREC2 *to silencing *ClREC2* expression. Bar = 1cm. B, The relative expression levels of chlorophyll*-*related genes in leaves infiltrated with pV190 or pV190-*ClREC2*. ClCHLI, ClCHLD, ClCHLE and ClCHLH was Magnesium-chelatase subunit ChlI, Magnesium-chelatase subunit ChlD, Magnesium-protoporphyrin IX monomethyl ester and Magnesium-chelatase subunit ChlH, respectively. C, Ultrastructure of chloroplasts in leaves infiltrated with pV190 or pV190-*ClREC2*. Cp, chloroplast; SG, starch granules; GT, granum thylakoid. Bar = 1µm. D, The chlorophyll and carotenoid contents in leaves infiltrated with pV190 or pV190-*ClREC2*. Chls, Chla, Chlb and Caro was the abbreviation of chlorophylls, chlorophyll a, chlorophyll b and carotenoid, respectively. E, The relative expression levels of* ClCREC2*, MEP- and CB-pathway genes in leaf infiltrated with pV190 or pV190-*ClREC2* plants. In B, D and E, the values are the means ± SDs, with n = 3. Asterisks indicate differences from the control plant (*P < 0.05, **P < 0.01, Student’s t test).

Supplementary Material 11: Figure S11. Dynamics analysis of the lycopene and violaxanthin contents, and the expression of *ClREC2* in fruit flesh watermelons with different flesh colors. Fruit lycopene content (A), violaxanthin content (B) and *ClREC2* expression level (C) were measured at 10, 20 and 32 days after pollination (DAP). Statistically significant differences were determined by one-way ANOVA; the different lower letters indicate significant differences according to Duncan’s test (P < 0.05). The raw data used for these analyses were previously reported [Dynamics analysis of the lycopene and violaxanthin contents, and the expression of *ClREC2* in fruit flesh watermelons with different flesh colors. Fruit lycopene content (A), violaxanthin content (B) and *ClREC2* expression level (C) were measured at 10, 20 and 32 days after pollination (DAP). Statistically significant differences were determined by one-way ANOVA; the different lower letters indicate significant differences according to Duncan’s test (*P* < 0.05). The raw data used for these analyses were previously reported (Ren et al. 2014).

Supplementary Material 12: Table S1 The data generated by PacBio and Illumina sequencing technology. Table S2 Characteristics of optical maps generated using the direct label and stain protocol. Table S3 Assembly statistics of the DR117, 97103v2, Charleston Gray and G42 watermelon genomes. Table S4 Predicted protein-coding genes in the assembled genome. Table S5 Description of watermelon accessions representing a core collection of *Citrullus*. Table S6 Representative evaluation of the core collection of watermelon. Table S7 Length distributions of SVs in different categories. Table S8 Selective sweeps among different subgroups. Table S9 Top 10 most significant signals for each trait identified in the SNP-GWAS and SV-GWAS. Table S10 Genetic variants and fruit shape phenotype data of different water melon accessions. Table S11 Summary of the two variants for fruit shape in 196 core watermelon accessions. Table S12 Primers used in the present study. Table S13 Key variations and phenotypes of flesh color in watermelon germplasms. Table S14 The sequence detail of P5.4k. Table S15 Details of RNA-seq. Table S16 354 DEGs for flesh color in the bule module.

## Data Availability

All data generated or analyzed during this study are included in this article and its supplementary information files. The raw sequence data have been deposited in the NCBI Sequence Read Archive (SRA) under BioProject PRJNA982968, PRJNA980840, PRJNA983768 and PRJNA983085.

## Abbreviations

ClLCYB: Lycopene β-cyclase
SNP: Single Nucleotide Polymorphism
QTL: Quantitative Trait Locus
SVs: Structural Variations
CNVs: Copy Number Variants
InDels: Insertions/Delections
LD: Linkage Disequilibrium
IBS: Identical-By-State
MEP: Methylerythritol 4-phosphate
CB: Carotenoid Biosynthesis
ClISPD: 2-C-methyl-D-erythritol 4-phosphate cytidylyltransferase
ClISPG: 4-Hydroxy-3-methylbut-2-en-1-yl diphosphate synthase
ClISPH: 4-Hydroxy-3-methylbut-2-enyl diphosphate reductas
ClIDI2: Isopentenyl diphosphate delta-isomerase I
ClGGPPS: Geranylgeranyl diphosphate synthase
ClPDS: Phytoene desaturase
ClBCH: β-Carotene hydroxyl-lase
ClCHRC: Plastoglobulin carotenoid-associated protein
GWAS: Genome-Wide Association Study
TRP: Tetratricopeptide Repeat Protein
ClREC: REDUCED CHLOROPLAST COVERAGE protein
qRT-QTL: Quantitative Reverse Transcription PCR
FPKM: Fragments per kilobase
ClPAP: Plastid lipid-associated protein
WGCNA: Weighted gene coexpression network analysis
ClDXS: 1-Deoxy-D-xylulose-5-phosphate synthase 2
ClISPE: 4-Diphosphocytidyl-2-C-methyl-D-erythritol kinase
ClISPF: 2-C-methyl-D-erythritol 2,4-cyclodiphosphate synthase
ClZ-ISO: Z-carotene isomerase
ClZDS: Z-carotene desaturase
ClCRTISO: Carotenoid isomerase
DEGs: Differentially Expressed Genes
ClGGR: Geranylgeranyl reductase
ClCBSX: CBS domain-containing protein
ClDXR/ClISPC: 1-Deoxy-D-xylulose 5-phosphate reductoisomerase
VIGS: Virus-induced gene silencing
RCP2: REDUCED CAROTENOID PIGMENTATION 2

## References

[CR1] Alkan C, Coe BP, Eichler EE. Genome structural variation discovery and genotyping. Nat Rev Genet. 2011;12(5):363–75. 21358748 10.1038/nrg2958 PMC4108431

[CR2] Arends D, Prins P, Jansen RC, Broman KW. R/qtl: high-throughput multiple QTL mapping. Bioinformatics. 2010;26(23):2990–2. 20966004 10.1093/bioinformatics/btq565 PMC2982156

[CR3] Arnon DI. Copper enzymes in isolated chloroplasts. Plant Physiol. 1949;24(1):1–15. 16654194 10.1104/pp.24.1.1 PMC437905

[CR4] Arumuganathan K, Earle E. Nuclear DNA content of some important plant species. Plant Mol Biol Report. 1991;9:208–18.

[CR5] Bang H, Kim S, Leskovar D, King S. Development of a codominant CAPS marker for allelic selection between canary yellow and red watermelon based on SNP in lycopene beta-cyclase (LCYB) gene. Mol Breeding. 2007;20(1):63–72.

[CR6] Bradbury PJ, Zhang Z, Kroon DE, Casstevens TM, Ramdoss Y, Buckler ES. TASSEL: software for association mapping of complex traits in diverse samples. Bioinformatics. 2007;23(19):2633–5. 17586829 10.1093/bioinformatics/btm308

[CR7] Branham S, Vexler L, Meir A, Tzuri G, Frieman Z, Levi A, Wechter WP, Tadmor Y, Gur A. Genetic mapping of a major codominant QTL associated with beta-carotene accumulation in watermelon. Mol Breed. 2017;37(12):1–3.28127252

[CR8] Bréhélin C, Kessler F. The plastoglobule: a bag full of lipid biochemistry tricks. Photochem Photobiol. 2008;84(6):1388–94. 19067960 10.1111/j.1751-1097.2008.00459.x

[CR9] Chaisson MJP, Sanders AD, Zhao XF, Malhotra A, Porubsky D, Rausch T, Gardner EJ, Rodriguez OL, Guo L, Collins RL, et al. Multi-platform discovery of haplotype-resolved structural variation in human genomes. Nat Commun. 2019;10:1784. 30992455 10.1038/s41467-018-08148-z PMC6467913

[CR10] Chang CC, Chow CC, Tellier LC, Vattikuti S, Purcell SM, Lee JJ. Second-generation PLINK: rising to the challenge of larger and richer datasets. Gigascience. 2015;4:7. 25722852 10.1186/s13742-015-0047-8 PMC4342193

[CR11] Chiang C, Layer RM, Faust GG, Lindberg MR, Rose DB, Garrison EP, Marth GT, Quinlan AR, Hall IM. SpeedSeq: ultra-fast personal genome analysis and interpretation. Nat Methods. 2015;12(10):966–8. 26258291 10.1038/nmeth.3505 PMC4589466

[CR12] Danecek P, Auton A, Abecasis G, Albers CA, Banks E, DePristo MA, Handsaker RE, Lunter G, Marth GT, Sherry ST, et al. The variant call format and VCFtools. Bioinformatics. 2011;27(15):2156–8. 21653522 10.1093/bioinformatics/btr330 PMC3137218

[CR13] De Beukelaer H, Davenport GF, Fack V. Core Hunter 3: flexible core subset selection. BMC Bioinformatics. 2018;19:203. 29855322 10.1186/s12859-018-2209-z PMC6092719

[CR14] Deng Y, Liu SC, Zhang YL, Tan JS, Li XP, Chu X, Xu BH, Tian Y, Sun YD, Li BS, et al. A telomere-to-telomere gap-free reference genome of watermelon and its mutation library provide important resources for gene discovery and breeding. Mol Plant. 2022;15(8):1268–84. 35746868 10.1016/j.molp.2022.06.010

[CR15] DePristo MA, Banks E, Poplin R, Garimella KV, Maguire JR, Hartl C, Philippakis AA, del Angel G, Rivas MA, Hanna M, et al. A framework for variation discovery and genotyping using next-generation DNA sequencing data. Nat Genet. 2011;43(5):491–8. 21478889 10.1038/ng.806 PMC3083463

[CR16] Dou JL, Zhao SJ, Lu XQ, He N, Zhang L, Ali A, Kuang HH, Liu WG. Genetic mapping reveals a candidate gene (ClFS1) for fruit shape in watermelon (Citrullus lanatus L.). Theor Appl Genet. 2018;131(4):947–58. 29362832 10.1007/s00122-018-3050-5

[CR17] Espley RV, Brendolise C, Chagné D, Kutty-Amma S, Green S, Volz R, Putterill J, Schouten HJ, Gardiner SE, Hellens RP, et al. Multiple repeats of a promoter segment causes transcription factor autoregulation in red apples. Plant Cell. 2009;21(1):168–83. 19151225 10.1105/tpc.108.059329 PMC2648084

[CR18] Falush D, Stephens M, Pritchard JK. Inference of population structure using multilocus genotype data: linked loci and correlated allele frequencies. Genetics. 2003;164(4):1567–87. 12930761 10.1093/genetics/164.4.1567 PMC1462648

[CR19] Fuentes RR, Chebotarov D, Duitama J, Smith S, De la Hoz JF, Mohiyuddin M, Wing RA, McNally KL, Tatarinova T, Grigoriev A, et al. Structural variants in 3000 rice genomes. Genome Res. 2019;29(5):870–80. 30992303 10.1101/gr.241240.118 PMC6499320

[CR20] Granato ISC, Galli G, Couto EGD, Souza MBE, Mendonca LF, Fritsche R. snpReady: a tool to assist breeders in genomic analysis. Mol Breeding. 2018;38(8):1–7.

[CR21] Guo SG, Zhang JG, Sun HH, Salse J, Lucas WJ, Zhang HY, Zheng Y, Mao LY, Ren Y, Wang ZW, et al. The draft genome of watermelon (Citrullus lanatus) and resequencing of 20 diverse accessions. Nat Genet. 2013;45(1):51–8. 23179023 10.1038/ng.2470

[CR22] Guo S, Zhao S, Sun H, Wang X, Wu S, Lin T, Ren Y, Gao L, Deng Y, Zhang J, et al. Resequencing of 414 cultivated and wild watermelon accessions identifies selection for fruit quality traits. Nat Genet. 2019;51(11):1616–23. 31676863 10.1038/s41588-019-0518-4

[CR23] Jeffares DC, Jolly C, Hoti M, Speed D, Shaw L, Rallis C, Balloux F, Dessimoz C, Bahler J, Sedlazeck FJ. Transient structural variations have strong effects on quantitative traits and reproductive isolation in fission yeast. Nat Commun. 2017;8:14061. 28117401 10.1038/ncomms14061 PMC5286201

[CR24] Ji GX, Liang CZ, Cai YF, Pan ZE, Meng ZG, Li YY, Jia YH, Miao YC, Pei XX, Gong WF, et al. A copy number variant at the HPDA-D12 locus confers compact plant architecture in cotton. New Phytol. 2021;229(4):2091–103. 33129229 10.1111/nph.17059

[CR25] Jie Z, Guoyi G, Shaogui G, Yi R, Haiying Z. YONG X: Fine mapping of the flesh color controlling genes in watermelon (Citrullus lanatus). Cucurbitaceae. 2014;2014:111.

[CR26] Kilambi HV, Kumar R, Sharma R, Sreelakshmi Y. Chromoplast-specific carotenoid-associated protein appears to be important for enhanced accumulation of carotenoids in tomato fruits. Plant Physiol. 2013;161(4):2085–101. 23400702 10.1104/pp.112.212191 PMC3613478

[CR27] Koren S, Walenz BP, Berlin K, Miller JR, Bergman NH, Phillippy AM. Canu: scalable and accurate long-read assembly via adaptive k-mer weighting and repeat separation. Genome Res. 2017;27(5):722–36. 28298431 10.1101/gr.215087.116 PMC5411767

[CR28] Larkin RM, Stefano G, Ruckle ME, Stavoe AK, Sinkler CA, Brandizzi F, Malmstrom CM, Osteryoung KW. REDUCED CHLOROPLAST COVERAGE genes from help to establish the size of the chloroplast compartment. P Natl Acad Sci USA. 2016;113(8):E1116–25. 10.1073/pnas.1515741113 PMC4776492 26862170

[CR29] Layer RM, Chiang C, Quinlan AR, Hall IM. LUMPY: a probabilistic framework for structural variant discovery. Genome Biol. 2014;15(6):1–9. 10.1186/gb-2014-15-6-r84 PMC4197822 24970577

[CR30] Legendre R, Kuzy J, McGregor C. Markers for selection of three alleles of ClSUN25–26–27a (Cla011257) associated with fruit shape in watermelon. Mol Breeding. 2020;40(2):1–3.

[CR31] Li H, Durbin R. Fast and accurate short read alignment with Burrows-Wheeler transform. Bioinformatics. 2009;25(14):1754–60. 19451168 10.1093/bioinformatics/btp324 PMC2705234

[CR32] Li L, Yuan H. Chromoplast biogenesis and carotenoid accumulation. Arch Biochem Biophys. 2013;539(2):102–9. 23851381 10.1016/j.abb.2013.07.002

[CR33] Li N, Shang J, Wang J, Zhou D, Ma S. Discovery of the genomic region and candidate genes of the Scarlet Red Flesh Color (Y^scr^ ) locus in watermelon (Citrullus Lanatus L.). Front Plant Sci. 2020;11:116. 32140168 10.3389/fpls.2020.00116 PMC7043143

[CR34] Li N, Shang J, Li N, Zhou D, Kong S, Wang J, Ma S. Accurate molecular identification for fruit shape in watermelon (Citrullus lanatus). Acta Horticulturae Sinica. 2021;48(7):1386–96.

[CR35] Li N, Kong S, Zhou D, Li N, Shang J, Wang J, Ma S. Mapping and validation of a new quantitative trait locus (QTL) for fruit size in watermelon (Citrullus lanatus). Sci Hortic-Amsterdam. 2023;318:112054.

[CR36] Liang M, Chen WJ, LaFountain AM, Liu YL, Peng F, Xia R, Bradshaw HD, Yuan YW. Taxon-specific, phased siRNAs underlie a speciation locus in monkeyflowers. Science. 2023;379(6632):576–82. 36758083 10.1126/science.adf1323 PMC10601778

[CR37] Liao NQ, Hu ZY, Li YY, Hao JF, Chen SN, Xue Q, Ma YY, Zhang KJ, Mahmoud A, Ali A, et al. Ethylene-responsive factor 4 is associated with the desirable rind hardness trait conferring cracking resistance in fresh fruits of watermelon. Plant Biotechnol J. 2020;18(4):1066–77. 31610078 10.1111/pbi.13276 PMC7061880

[CR38] Lipka AE, Tian F, Wang QS, Peiffer J, Li M, Bradbury PJ, Gore MA, Buckler ES, Zhang ZW. GAPIT: genome association and prediction integrated tool. Bioinformatics. 2012;28(18):2397–9. 22796960 10.1093/bioinformatics/bts444

[CR39] Liu M, Liang ZL, Aranda MA, Hong N, Liu LM, Kang BS, Gu QS. A cucumber green mottle mosaic virus vector for virus-induced gene silencing in cucurbit plants. Plant Methods. 2020;16(1):9. 32025236 10.1186/s13007-020-0560-3 PMC6996188

[CR40] Liu S, Gao Z, Wang X, Luan F, Dai Z, Yang Z, Zhang Q. Nucleotide variation in the phytoene synthase (ClPsy1) gene contributes to golden flesh in watermelon (Citrullus lanatus L.). Theor Appl Genet. 2022;135(1):185–200. 34633472 10.1007/s00122-021-03958-0

[CR41] Love MI, Huber W, Anders S. Moderated estimation of fold change and dispersion for RNA-seq data with DESeq2. Genome Biol. 2014;15(12):550. 25516281 10.1186/s13059-014-0550-8 PMC4302049

[CR42] Ma S, Liu J: Decriptors and data standard for watermelon (Citrullus Schrad.): Chinese Agriculture Press; 2005.

[CR43] Marcais G, Kingsford C. A fast, lock-free approach for efficient parallel counting of occurrences of k-mers. Bioinformatics. 2011;27(6):764–70. 21217122 10.1093/bioinformatics/btr011 PMC3051319

[CR44] Martin A, Troadec C, Boualem A, Rajab M, Fernandez R, Morin H, Pitrat M, Dogimont C, Bendahmane A. A transposon-induced epigenetic change leads to sex determination in melon. Nature. 2009;461(7267):1135–8. 19847267 10.1038/nature08498

[CR45] Pan YP, Wang YH, McGregor C, Liu S, Luan FS, Gao ML, Weng YQ. Genetic architecture of fruit size and shape variation in cucurbits: a comparative perspective. Theor Appl Genet. 2020;133(1):1–21. 31768603 10.1007/s00122-019-03481-3

[CR46] Quevillon E, Silventoinen V, Pillai S, Harte N, Mulder N, Apweiler R, Lopez R. InterProScan: protein domains identifier. Nucleic Acids Res. 2005;33:W116–20. 15980438 10.1093/nar/gki442 PMC1160203

[CR47] Ren Y, McGregor C, Zhang Y, Gong GY, Zhang HY, Guo SG, Sun HH, Cai WT, Zhang J, Xu Y. An integrated genetic map based on four mapping populations and quantitative trait loci associated with economically important traits in watermelon (Citrullus lanatus). Bmc Plant Biol. 2014;14:33. 24443961 10.1186/1471-2229-14-33 PMC3898567

[CR48] Ren Y, Guo SG, Zhang J, He HJ, Sun HH, Tian SW, Gong GY, Zhang HY, Levi A, Tadmor Y, et al. A tonoplast sugar transporter underlies a sugar accumulation QTL in watermelon. Plant Physiol. 2018;176(1):836–50. 29118248 10.1104/pp.17.01290 PMC5761790

[CR49] Ren Y, Sun H, Zong M, Guo S, Ren Z, Zhao J, Li M, Zhang J, Tian S, Wang J, et al. Localization shift of a sugar transporter contributes to phloem unloading in sweet watermelons. New Phytol. 2020;227(6):1858–71. 32453446 10.1111/nph.16659

[CR50] Ren Y, Li M, Guo S, Sun H, Zhao J, Zhang J, Liu G, He H, Tian S, Yu Y, et al. Evolutionary gain of oligosaccharide hydrolysis and sugar transport enhanced carbohydrate partitioning in sweet watermelon fruits. Plant Cell. 2021;33(5):1554–73. 33570606 10.1093/plcell/koab055 PMC8254481

[CR51] Renner SS, Sousa A, Chomicki G. Chromosome numbers, Sudanese wild forms, and classification of the watermelon genus Citrullus, with 50 names allocated to seven biological species. Taxon. 2017;66(6):1393–405.

[CR52] Renner SS, Wu S, Pérez-Escobar OA, Silber MV, Fei ZJ, Chomicki G. A chromosome-level genome of a Kordofan melon illuminates the origin of domesticated watermelons. P Natl Acad Sci USA. 2021;118(23):e2101486118. 10.1073/pnas.2101486118 PMC8201767 34031154

[CR53] Robinson JT, Thorvaldsdottir H, Winckler W, Guttman M, Lander ES, Getz G, Mesirov JP. Integrative genomics viewer. Nat Biotechnol. 2011;29(1):24–6. 21221095 10.1038/nbt.1754 PMC3346182

[CR54] Sandlin K, Prothro J, Heesacker A, Khalilian N, Okashah R, Xiang W, Bachlava E, Caldwell DG, Taylor CA, Seymour DK, et al. Comparative mapping in watermelon [Citrullus lanatus (Thunb.) Matsum. et Nakai]. Theor Appl Genet. 2012;125(8):1603–18. 22875176 10.1007/s00122-012-1938-z

[CR55] Simao FA, Waterhouse RM, Ioannidis P, Kriventseva EV, Zdobnov EM. BUSCO: assessing genome assembly and annotation completeness with single-copy orthologs. Bioinformatics. 2015;31(19):3210–2. 26059717 10.1093/bioinformatics/btv351

[CR56] Stanley LE, Ding BQ, Sun W, Mou FJ, Hill C, Chen SL, Yuan YW. A tetratricopeptide repeat protein regulates carotenoid biosynthesis and chromoplast development in Monkeyflowers (Mimulus). Plant Cell. 2020;32(5):1536–55. 32132132 10.1105/tpc.19.00755 PMC7203930

[CR57] Sun SL, Zhou YS, Chen J, Shi JP, Zhao HM, Zhao HN, Song WB, Zhang M, Cui Y, Dong XM, et al. Extensive intraspecific gene order and gene structural variations between Mo17 and other maize genomes. Nat Genet. 2018;50(9):1289–95. 30061735 10.1038/s41588-018-0182-0

[CR58] Tian SW, Jiang LJ, Gao Q, Zhang J, Zong M, Zhang HY, Ren Y, Guo SG, Gong GY, Liu F, et al. Efficient CRISPR/Cas9-based gene knockout in watermelon. Plant Cell Rep. 2017;36(3):399–406. 27995308 10.1007/s00299-016-2089-5

[CR59] Trapnell C, Williams BA, Pertea G, Mortazavi A, Kwan G, van Baren MJ, Salzberg SL, Wold BJ, Pachter L. Transcript assembly and quantification by RNA-Seq reveals unannotated transcripts and isoform switching during cell differentiation. Nat Biotechnol. 2010;28(5):511–5. 20436464 10.1038/nbt.1621 PMC3146043

[CR60] Walker BJ, Abeel T, Shea T, Priest M, Abouelliel A, Sakthikumar S, Cuomo CA, Zeng QD, Wortman J, Young SK, et al. Pilon: an integrated tool for comprehensive microbial variant detection and genome assembly improvement. Plos One. 2014;9(11):e112963. 25409509 10.1371/journal.pone.0112963 PMC4237348

[CR61] Wang WS, Mauleon R, Hu ZQ, Chebotarov D, Tai SS, Wu ZC, Li M, Zheng TQ, Fuentes RR, Zhang F, et al. Genomic variation in 3,010 diverse accessions of Asian cultivated rice. Nature. 2018;557(7703):43–9. 29695866 10.1038/s41586-018-0063-9 PMC6784863

[CR62] Wehner TC. Gene list for watermelon 2012. Cucurbit Genet Cooperative Rep. 2012;36:40–64.

[CR63] Wu YH, Bhat PR, Close TJ, Lonardi S. Efficient and accurate construction of genetic linkage maps from the minimum spanning tree of a graph. Plos Genet. 2008;4:e1000212. 18846212 10.1371/journal.pgen.1000212 PMC2556103

[CR64] Wu S, Wang X, Reddy U, Sun H, Bao K, Gao L, Mao L, Patel T, Ortiz C, Abburi VL, et al. Genome of “Charleston Gray”, the principal American watermelon cultivar, and genetic characterization of 1,365 accessions in the U.S. National Plant Germplasm System watermelon collection. Plant Biotechnol J. 2019;17(12):2246–58. 31022325 10.1111/pbi.13136 PMC6835170

[CR65] Wu S, Sun HH, Gao L, Branham S, McGregor C, Renner SS, Xu Y, Kousik C, Wechter WP, Levi A, et al. A Citrullus genus super-pangenome reveals extensive variations in wild and cultivated watermelons and sheds light on watermelon evolution and domestication. Plant Biotechnol J. 2023;21(10):1926–8. 37490004 10.1111/pbi.14120 PMC10502741

[CR66] Xiao H, Jiang N, Schaffner E, Stockinger EJ, van der Knaap E. A retrotransposon-mediated gene duplication underlies morphological variation of tomato fruit. Science. 2008;319(5869):1527–30. 18339939 10.1126/science.1153040

[CR67] Xu N, Xu H, Xu ZJ, Li FC, Xu Q. Introgression of a complex genomic structural variation causes hybrid male sterility in GJ rice (Oryza sativa L.) subspecies. Int J Molec Sci. 2022;23(21):12804. 36361593 10.3390/ijms232112804 PMC9656383

[CR68] Yi L, Zhou W, Zhang Y, Chen Z, Wu N, Wang Y, Dai Z. Genetic mapping of a single nuclear locus determines the white flesh color in watermelon (Citrullus lanatus L.). Front Plant Sci. 2023;14:1090009. 36824206 10.3389/fpls.2023.1090009 PMC9941332

[CR69] Ytterberg AJ, Peltier JB, van Wijk KJ. Protein profiling of plastoglobules in chloroplasts and chromoplasts. A surprising site for differential accumulation of metabolic enzymes. Plant Physiol. 2006;140(3):984–97. 16461379 10.1104/pp.105.076083 PMC1400577

[CR70] Yuan PL, Umer MJ, He N, Zhao SJ, Lu XQ, Zhu HJ, Gong CS, Diao WN, Gebremeskel H, Kuang HH, et al. Transcriptome regulation of carotenoids in five flesh-colored watermelons (Citrullus lanatus). Bmc Plant Biol. 2021;21:230. 33910512 10.1186/s12870-021-02965-z PMC8082968

[CR71] Zdobnov EM, Apweiler R. InterProScan - an integration platform for the signature-recognition methods in InterPro. Bioinformatics. 2001;17(9):847–8. 11590104 10.1093/bioinformatics/17.9.847

[CR72] Zhang J, Guo SG, Ren Y, Zhang HY, Gong GY, Zhou M, Wang GZ, Zong M, He HJ, Liu F, et al. High-level expression of a novel chromoplast phosphate transporter ClPHT4;2 is required for flesh color development in watermelon. New Phytol. 2017;213(3):1208–21. 27787901 10.1111/nph.14257

[CR73] Zhang C, Dong SS, Xu JY, He WM, Yang TL. PopLDdecay: a fast and effective tool for linkage disequilibrium decay analysis based on variant call format files. Bioinformatics. 2019;35(10):1786–8. 30321304 10.1093/bioinformatics/bty875

[CR74] Zhang J, Sun H, Guo S, Ren Y, Li M, Wang J, Zhang H, Gong G, Xu Y. Decreased protein abundance of lycopene beta-cyclase contributes to red flesh in domesticated watermelon. Plant Physiol. 2020;183(3):1171–83. 32321841 10.1104/pp.19.01409 PMC7333704

[CR75] Zheng JS, Hong K, Zeng LJ, Wang L, Kang SJ, Qu MH, Dai JR, Zou LY, Zhu LX, Tang ZP, et al. Karrikin signaling acts parallel to and additively with strigolactone signaling to regulate rice mesocotyl elongation in darkness. Plant Cell. 2020;32(9):2780–805. 32665307 10.1105/tpc.20.00123 PMC7474294

