# Supplementary figures and images for: Multi-omics analyses unveil dual genetic loci governing four distinct watermelon flesh color phenotypes
Source: Mol Hortic. 2025 May 14;5:46. doi: 10.1186/s43897-025-00166-y (PMC12077075; doi:10.1186/s43897-025-00166-y)

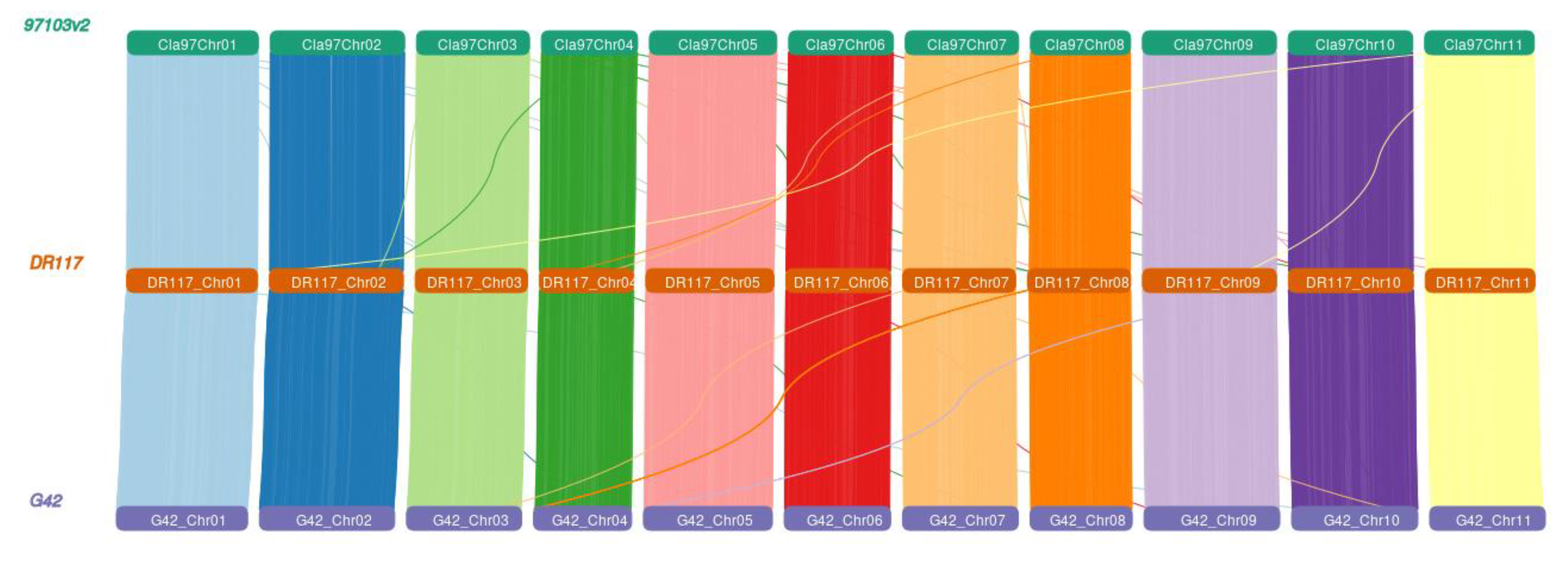

Supplement: Supplementary file 1 — Supplementary Material 1: Figure S1. Eleven superscaffolds (chromosomes) of the DR117 genome corresponding to 11 chromosomes of the 97103v2 and G42 genomes. [file 43897_2025_166_MOESM1_ESM.jpg]

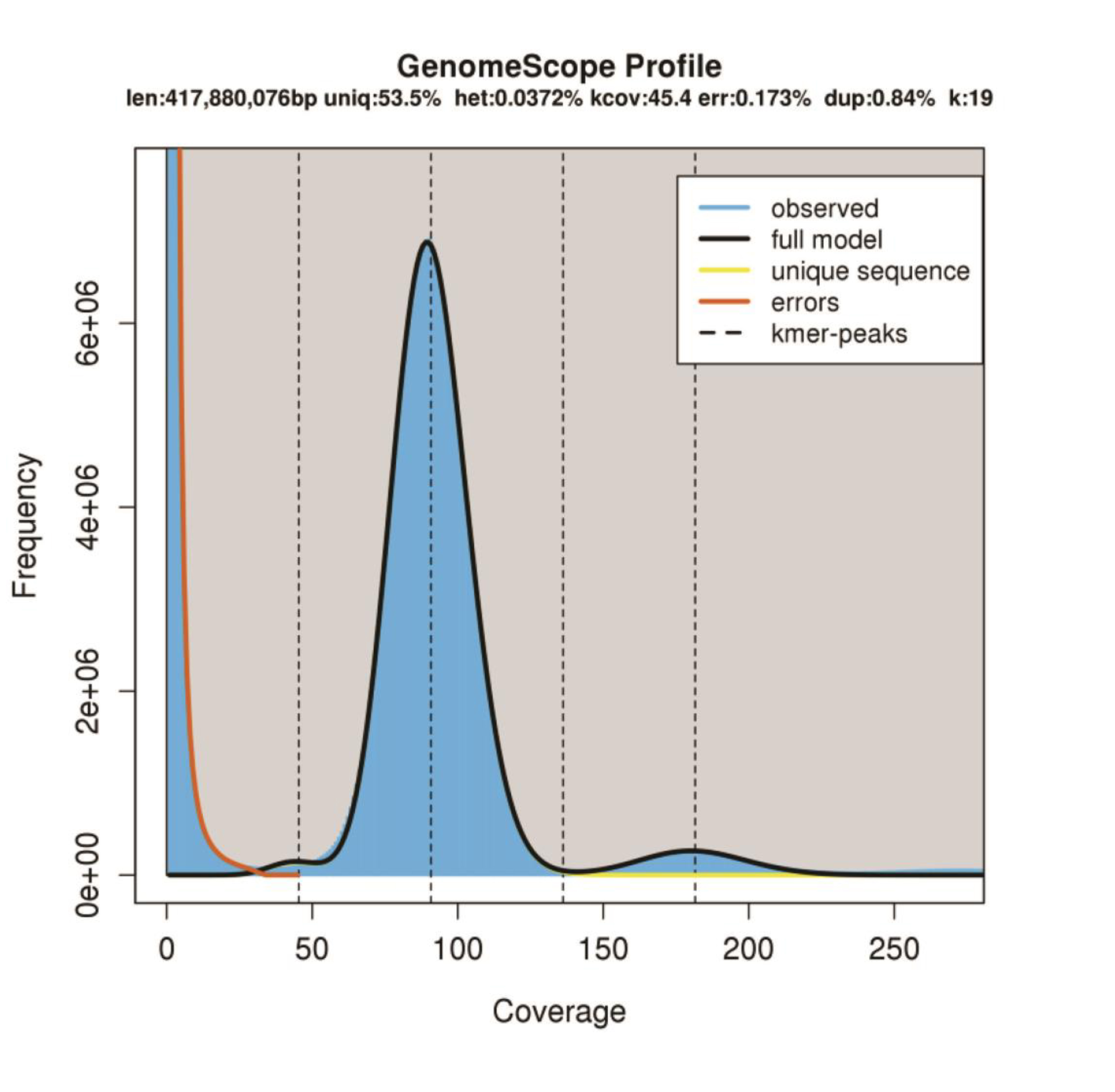

Supplement: Supplementary file 2 — Supplementary Material 2: Figure S2. K-mer analysis of the watermelon genome using GenomeScope. [file 43897_2025_166_MOESM2_ESM.jpg]

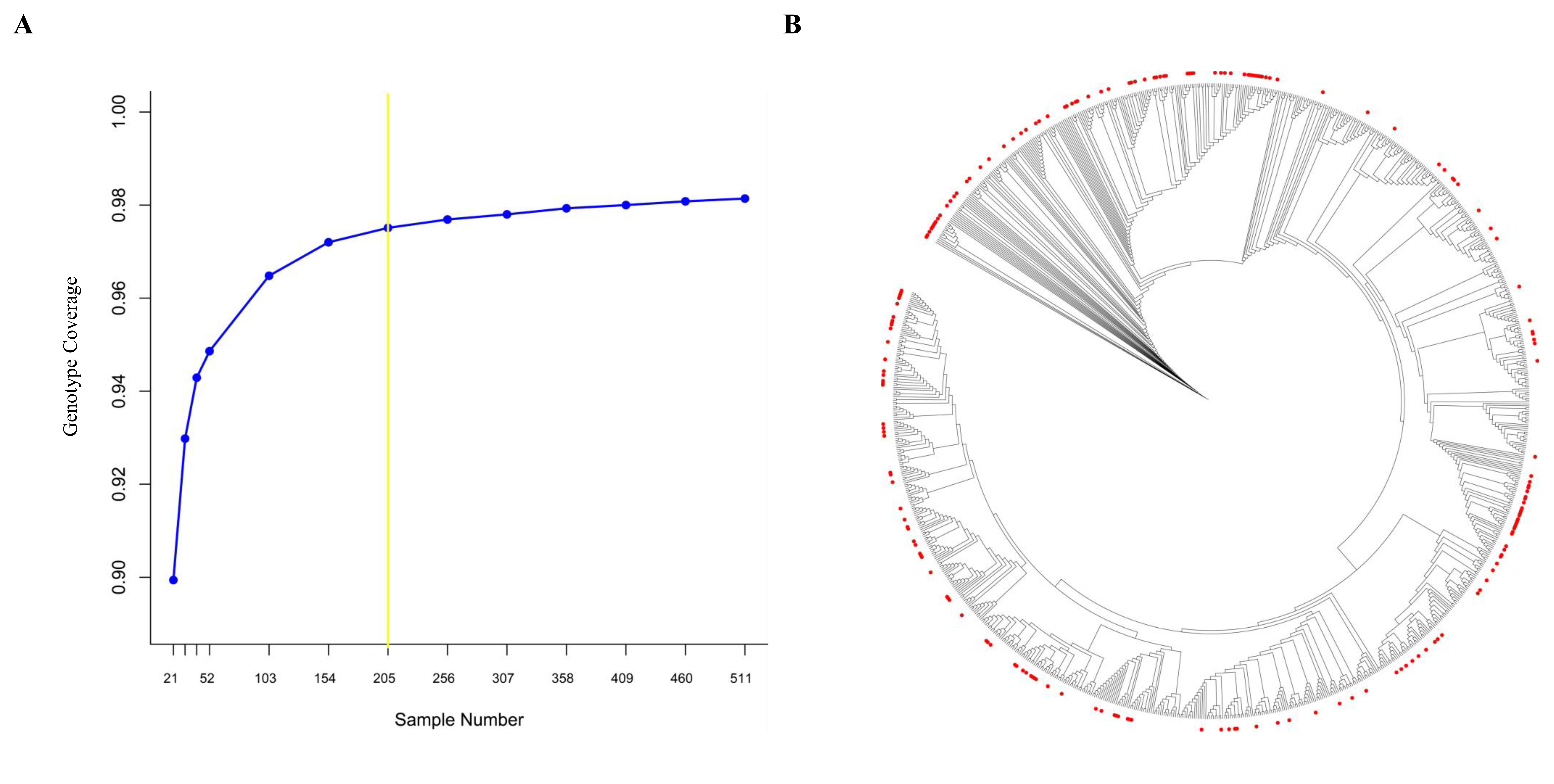

Supplement: Supplementary file 3 — Supplementary Material 3: Figure S3. Development and evaluation of the watermelon core collection. A, Coverage of allelic diversity versus number of selected accessions analyzed. B, Neighbor-joining phylogenetic trees of 1022 Citrullus accessions. The core collections are indicated by red dots. [file 43897_2025_166_MOESM3_ESM.jpg]

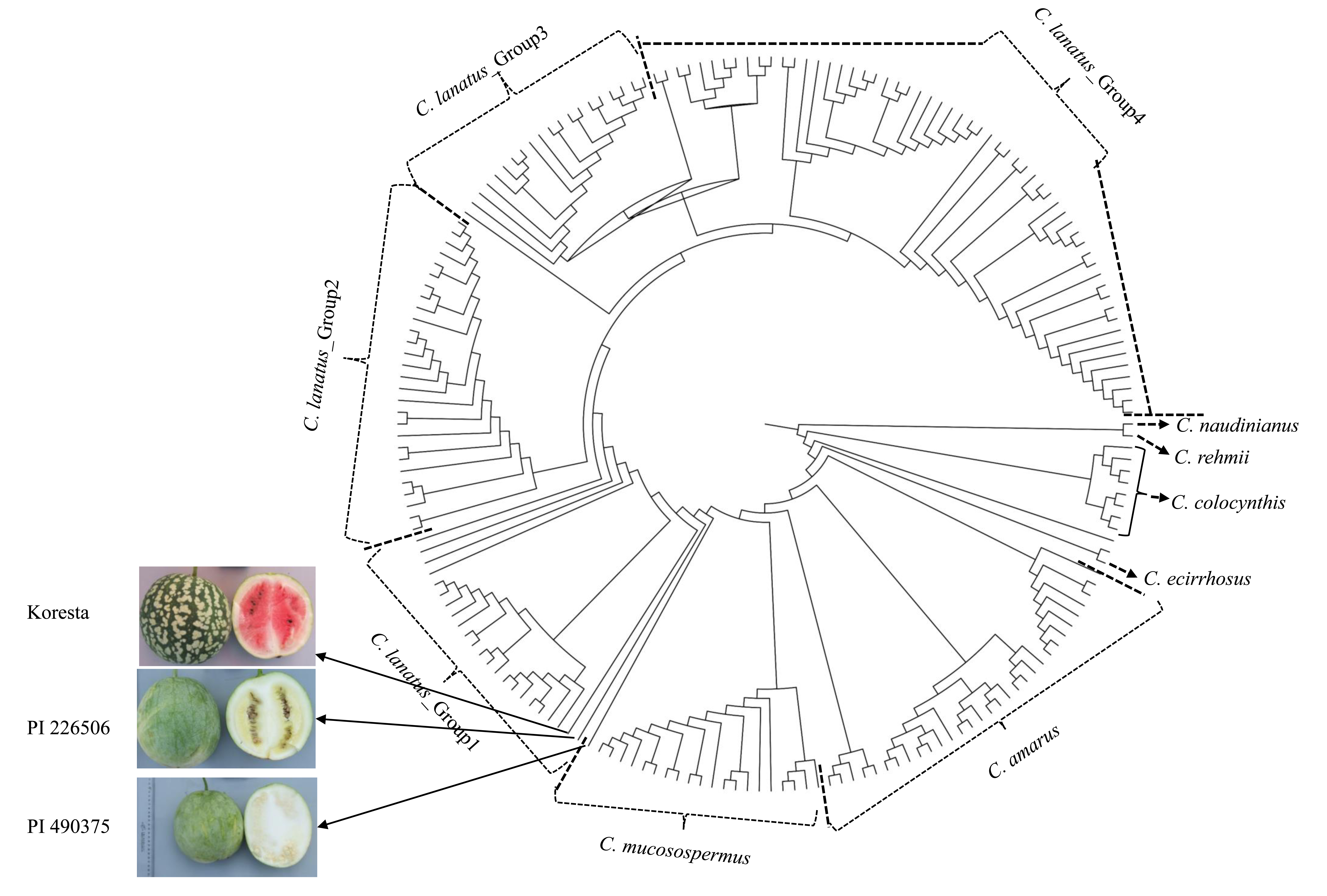

Supplement: Supplementary file 4 — Supplementary Material 4: Figure S4. Neighbor-joining phylogenetic trees of Citrullus accessions. Two C. lanatus accessions on the deepest branch of the C. lanatus clade and one C. mucosospermus accession on the lowest branch of the C. mucosospermus clade are indicated by the arrow. [file 43897_2025_166_MOESM4_ESM.jpg]

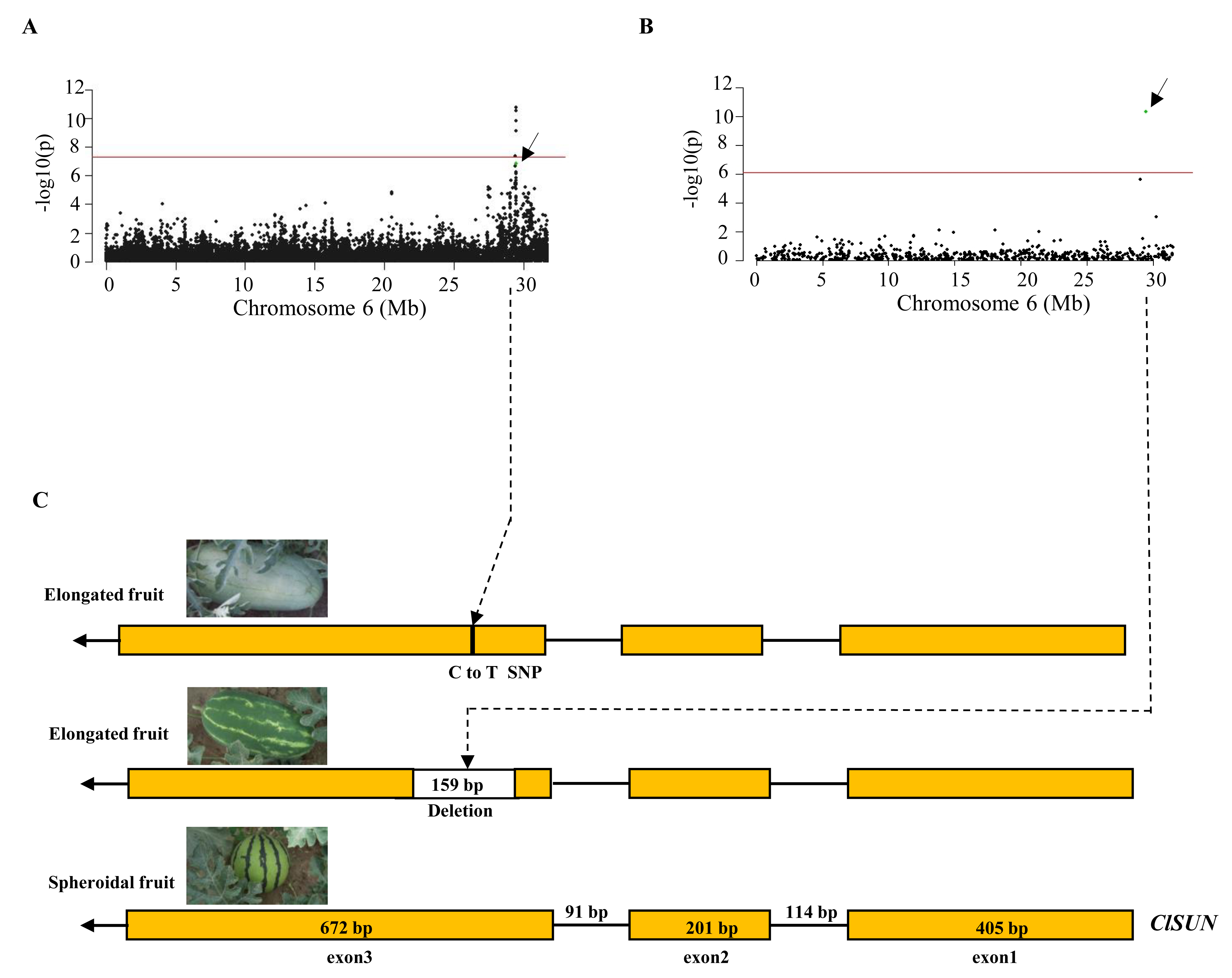

Supplement: Supplementary file 5 — Supplementary Material 5: Figure S5. SNP-GWAS and SV-GWAS identify loci of fruit shape in watermelon. A, SNP-GWAS for fruit shape. B, SV-GWAS for fruit shape. C, The location of causative variants in the candidate gene ClSUN related to fruit shape. The horizontal red lines in A-B indicate the genome-wide threshold of GWAS signals. [file 43897_2025_166_MOESM5_ESM.jpg]

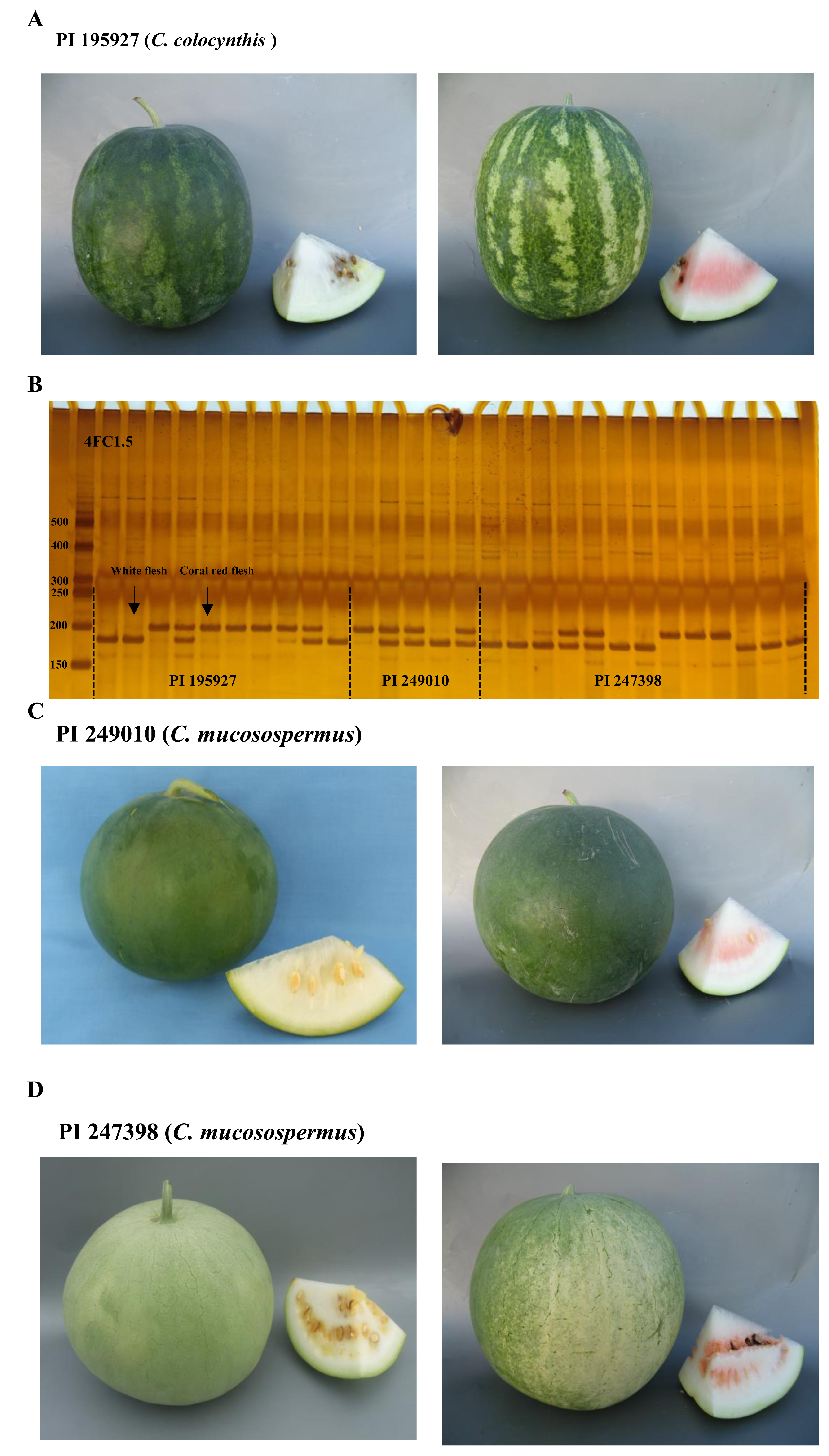

Supplement: Supplementary file 6 — Supplementary Material 6: Figure S6. The phenotypes and genotypes of C. colocynthis accession and two C. mucosospermus accessions with separation of flesh color. A, The fruit and flesh of PI 195927. B, Enzyme digestion products after PCR amplification by primer 4FC1.5. C, The fruit and flesh of PI 249010. D, The fruit and flesh of PI 247398. [file 43897_2025_166_MOESM6_ESM.jpg]

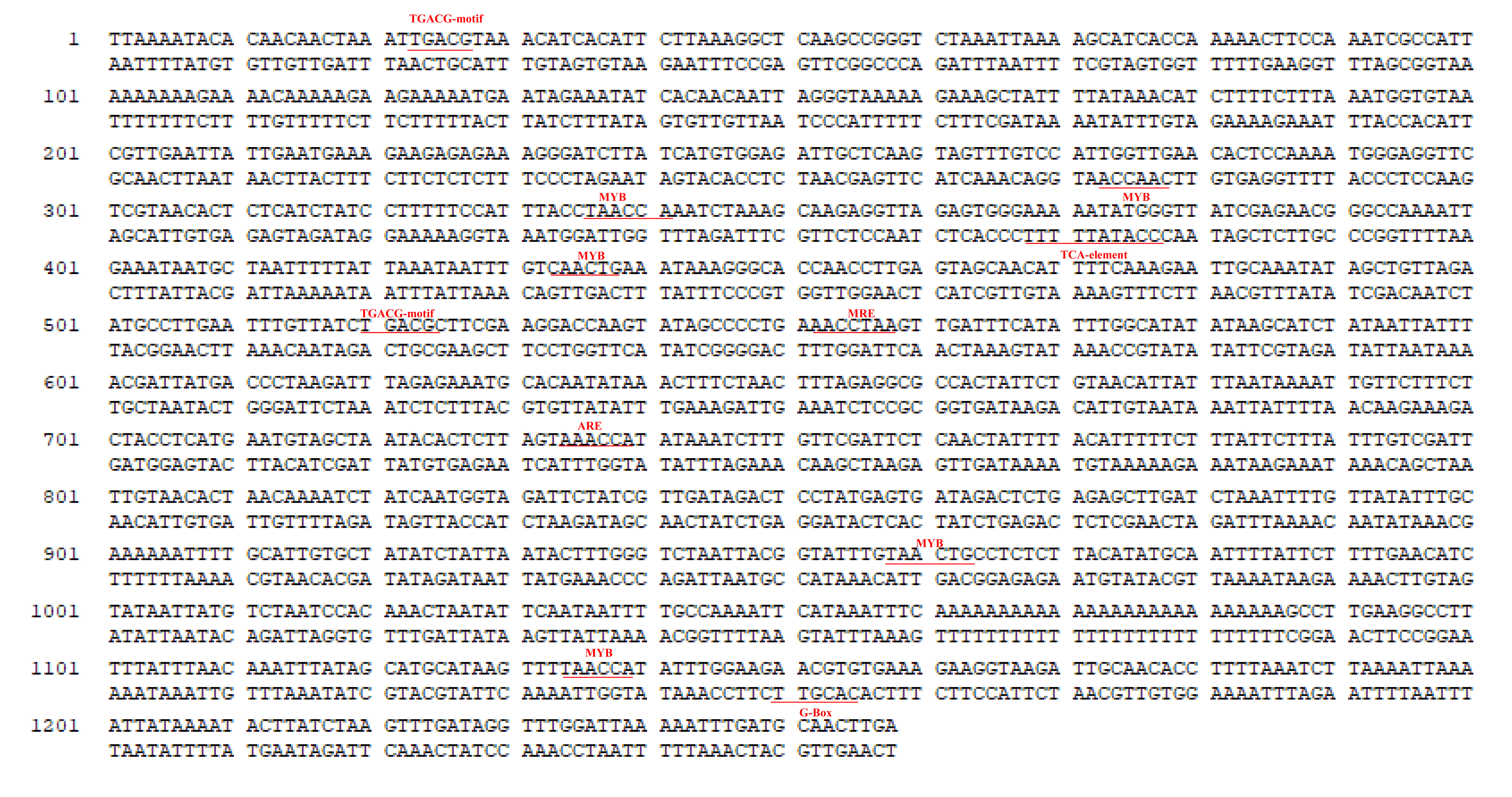

Supplement: Supplementary file 7 — Supplementary Material 7: Figure S7. Nucleotide sequence of the 1263 bp with putative cis-acting regulatory elements shown. The important putative cis acting regulatory elements are underlined with red lines. The “TGACG-motif” is a cis-acting regulatory element involved in the MeJA-responsiveness. The “TCA-element” is a cis-acting element involved in salicylic acid responsiveness. The “MRE” is a MYB binding site involved in light responsiveness. The “ARE” is a cis-acting regulatory element essential for the anaerobic induction. The “G-box” is a cis-acting regulatory element involved in light responsiveness. [file 43897_2025_166_MOESM7_ESM.jpg]

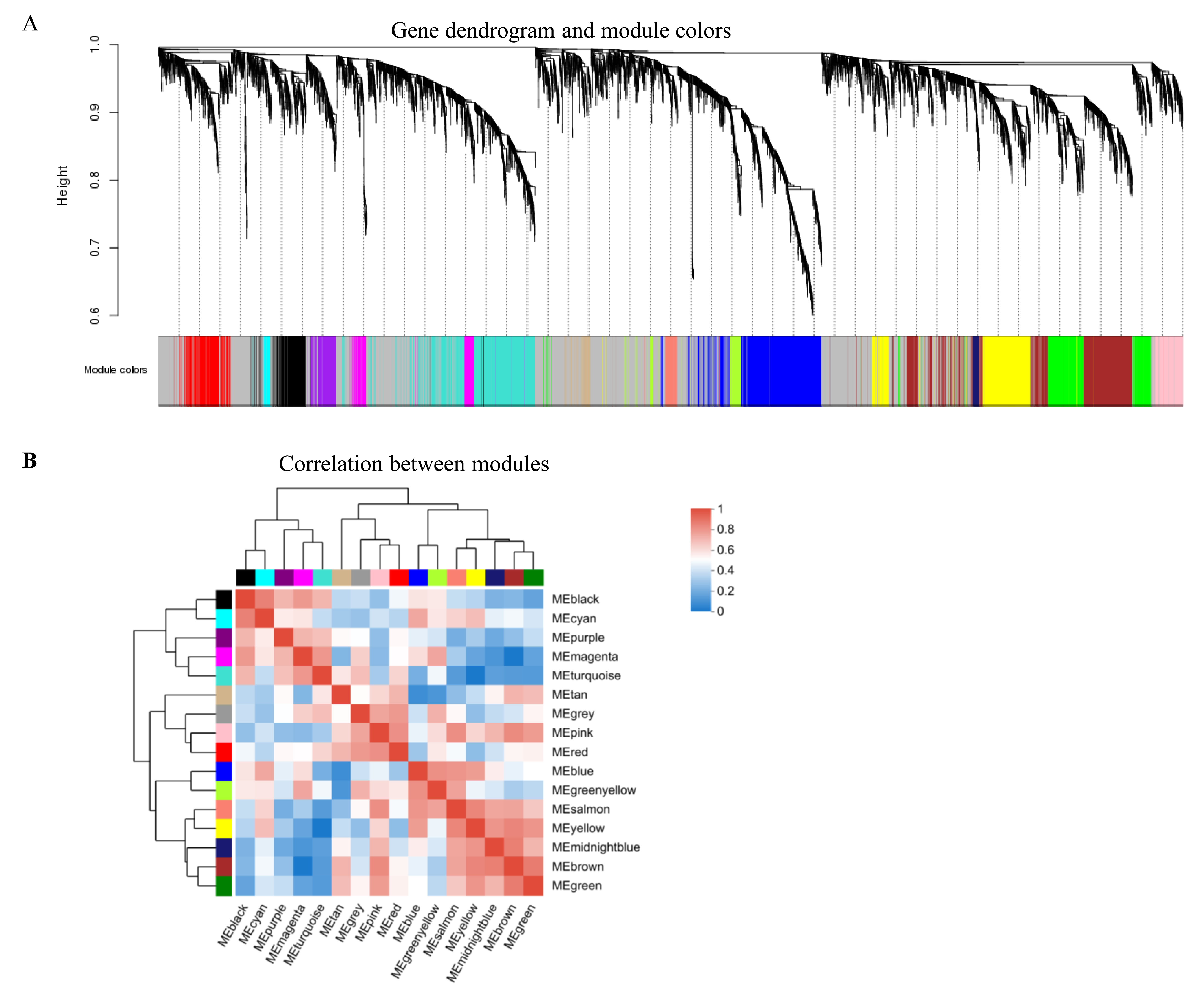

Supplement: Supplementary file 8 — Supplementary Material 8: Figure S8. Cluster dendrogram of genes in watermelon. A, Cluster dendrogram of genes and corresponding modules. Each color represents a module, and the gray color represents genes not included in any module. B, Heatmap of the correlations between modules. [file 43897_2025_166_MOESM8_ESM.jpg]

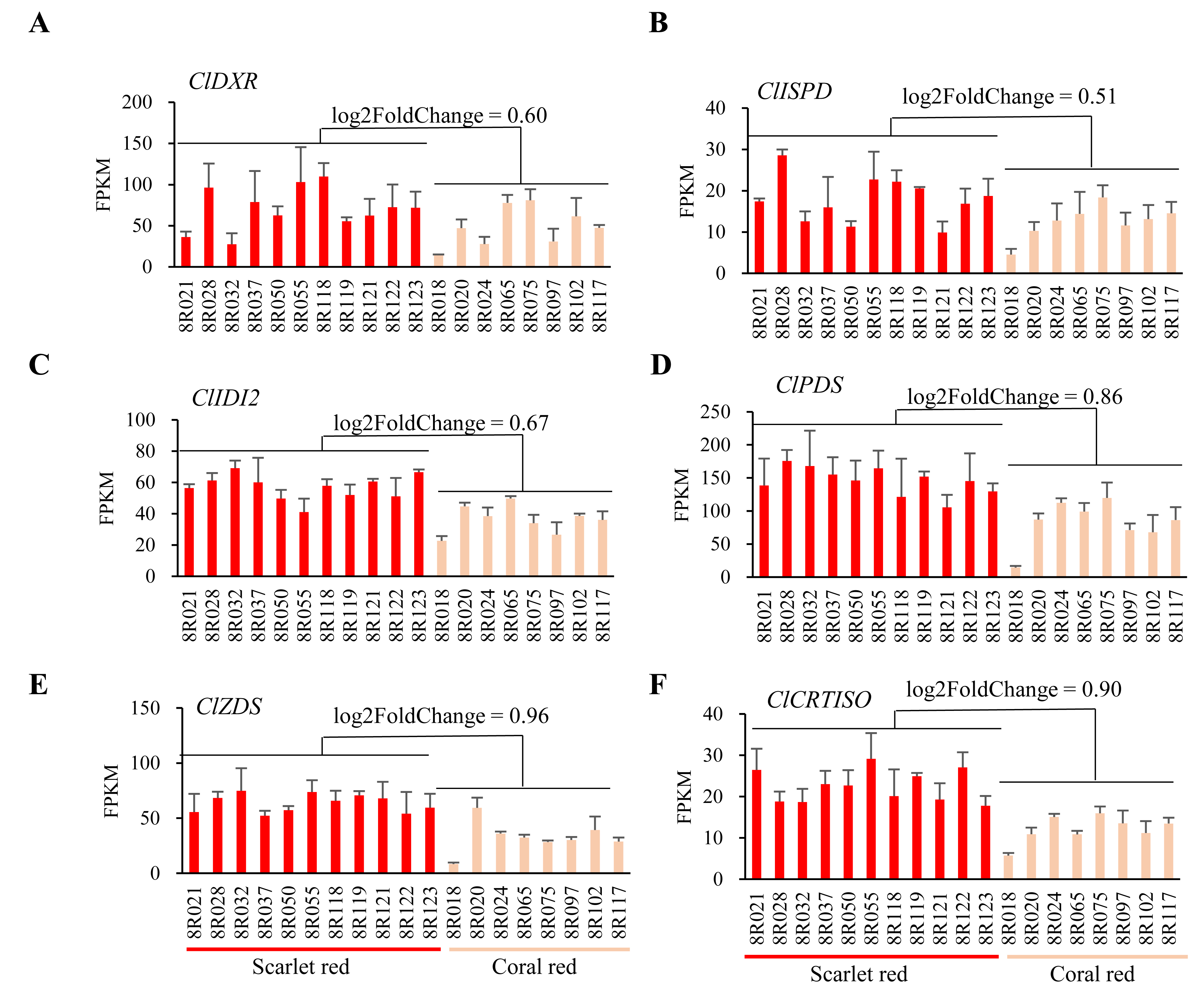

Supplement: Supplementary file 9 — Supplementary Material 9: Figure S9. The gene expression of ClDXR, ClISPD, ClIDI2, ClPDS, CZDS and ClCRTISO in 11 scarlet red and 8 coral red fleshed watermelons. FPKM of ClDXR (A), ClISPD (B), ClIDI2 (C), ClPDS (D), ClZDS (E), ClCRTISO (F) in 11 scarlet red and 8 coral red fleshed watermelon, respectively. The values are the means ± SDs, with n = 3. [file 43897_2025_166_MOESM9_ESM.jpg]

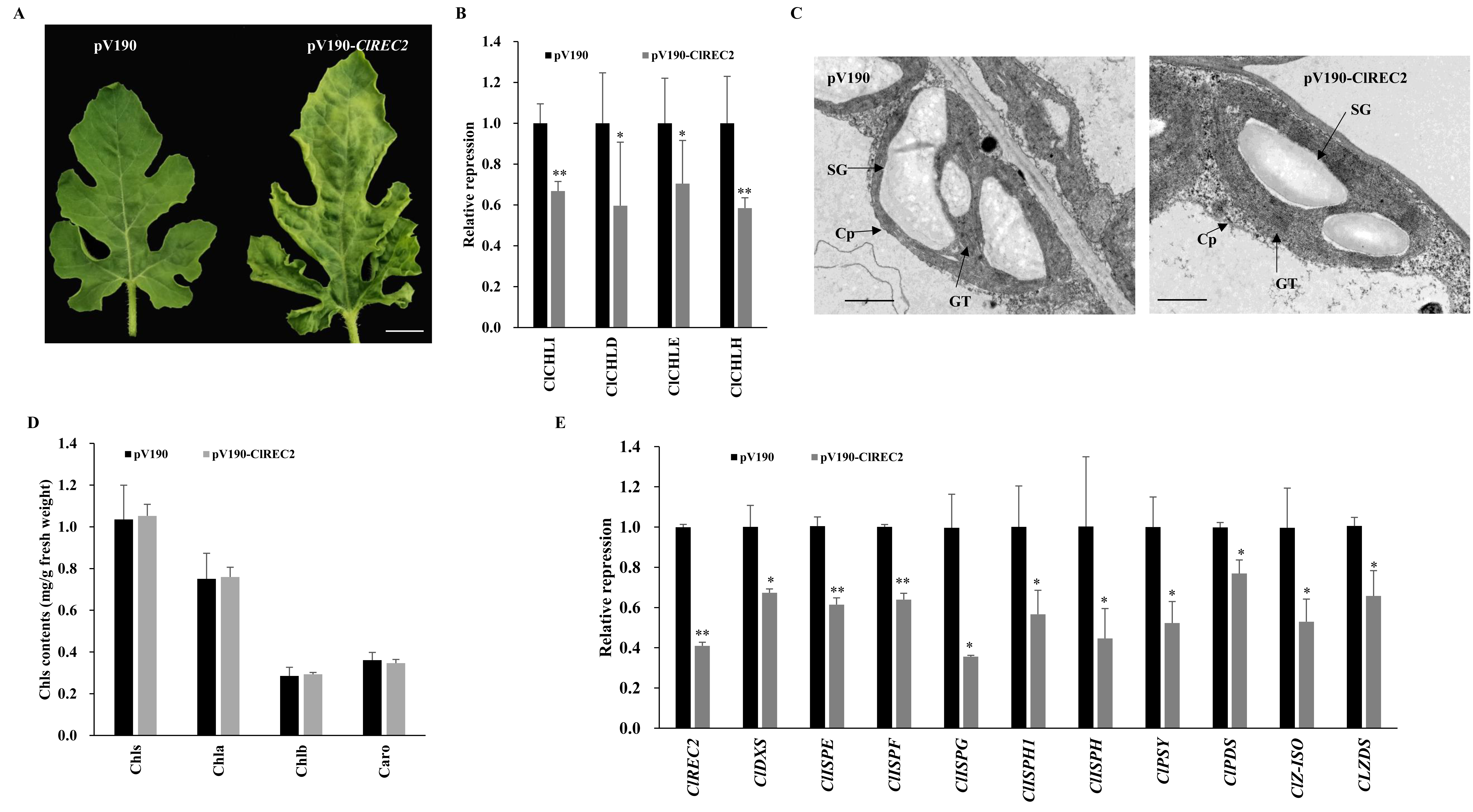

Supplement: Supplementary file 10 — Supplementary Material 10: Figure S10. Silencing ClREC2 in watermelon using the VIGS vector pV190. A, Watermelon leaf was infiltrated with the empty vector pV190 served as a control and another leaf was infiltrated with the vector pV190-ClREC2 to silencing ClREC2 expression. Bar = 1cm. B, The relative expression levels of chlorophyll-related genes in leaves infiltrated with pV190 or pV190-ClREC2. ClCHLI, ClCHLD, ClCHLE and ClCHLH was Magnesium-chelatase subunit ChlI, Magnesium-chelatase subunit ChlD, Magnesium-protoporphyrin IX monomethyl ester and Magnesium-chelatase subunit ChlH, respectively. C, Ultrastructure of chloroplasts in leaves infiltrated with pV190 or pV190-ClREC2. Cp, chloroplast; SG, starch granules; GT, granum thylakoid. Bar = 1µm. D, The chlorophyll and carotenoid contents in leaves infiltrated with pV190 or pV190-ClREC2. Chls, Chla, Chlb and Caro was the abbreviation of chlorophylls, chlorophyll a, chlorophyll b and carotenoid, respectively. E, The relative expression levels of ClCREC2, MEP- and CB-pathway genes in leaf infiltrated with pV190 or pV190-ClREC2 plants. In B, D and E, the values are the means ± SDs, with n = 3. Asterisks indicate differences from the control plant (*P < 0.05, **P < 0.01, Student’s t test). [file 43897_2025_166_MOESM10_ESM.jpg]

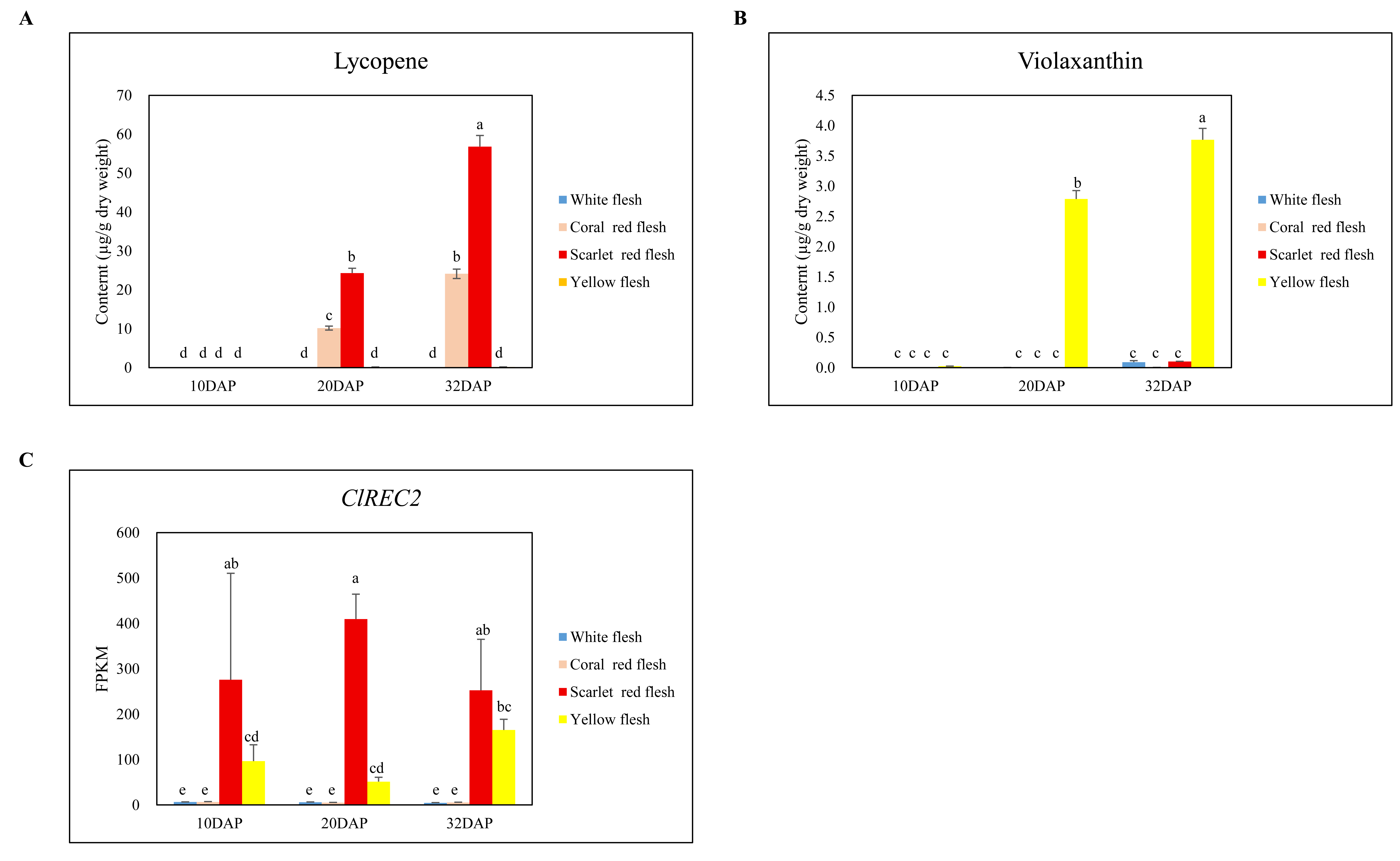

Supplement: Supplementary file 11 — Supplementary Material 11: Figure S11. Dynamics analysis of the lycopene and violaxanthin contents, and the expression of ClREC2 in fruit flesh watermelons with different flesh colors. Fruit lycopene content (A), violaxanthin content (B) and ClREC2 expression level (C) were measured at 10, 20 and 32 days after pollination (DAP). Statistically significant differences were determined by one-way ANOVA; the different lower letters indicate significant differences according to Duncan’s test (P < 0.05). The raw data used for these analyses were previously reported [Dynamics analysis of the lycopene and violaxanthin contents, and the expression of ClREC2 in fruit flesh watermelons with different flesh colors. Fruit lycopene content (A), violaxanthin content (B) and ClREC2 expression level (C) were measured at 10, 20 and 32 days after pollination (DAP). Statistically significant differences were determined by one-way ANOVA; the different lower letters indicate significant differences according to Duncan’s test (P < 0.05). The raw data used for these analyses were previously reported (Ren et al. 2014). [file 43897_2025_166_MOESM11_ESM.jpg]
